# Time varying methods to infer extremes in dengue transmission dynamics

**DOI:** 10.1371/journal.pcbi.1008279

**Published:** 2020-10-12

**Authors:** Jue Tao Lim, Yiting Han, Borame Sue Lee Dickens, Lee Ching Ng, Alex R. Cook

**Affiliations:** 1 Saw Swee Hock School of Public Health, National University of Singapore and National University Health System, Singapore; 2 School of Pharmacy, Fudan University, Shanghai, China; 3 Environmental Health Institute, National Environmental Agency, Singapore; University of Notre Dame, UNITED STATES

## Abstract

Dengue is an arbovirus affecting global populations. Frequent outbreaks occur, especially in equatorial cities such as Singapore, where year-round tropical climate, large daily influx of travelers and population density provide the ideal conditions for dengue to transmit. Little work has, however, quantified the peaks of dengue outbreaks, when health systems are likely to be most stretched. Nor have methods been developed to infer differences in exogenous factors which lead to the rise and fall of dengue case counts across extreme and non-extreme periods. In this paper, we developed time varying extreme mixture (tvEM) methods to account for the temporal dependence of dengue case counts across extreme and non-extreme periods. This approach permits inference of differences in climatic forcing across non-extreme and extreme periods of dengue case counts, quantification of their temporal dependence as well as estimation of thresholds with associated uncertainties to determine dengue case count extremities. Using tvEM, we found no evidence that weather affects dengue case counts in the near term for non-extreme periods, but that it has non-linear and mixed signals in influencing dengue through tvEM parameters in the extreme periods. Using the most appropriate tvEM specification, we found that a threshold at the 70^*th*^ (95% credible interval 41.1, 83.8) quantile is optimal, with extreme events of 526.6, 1052.2 and 1183.6 weekly case counts expected at return periods of 5, 50 and 75 years. Weather parameters at a 1% scaled increase was found to decrease the long-run expected case counts, but larger increases would lead to a drastic expected rise from the baseline correspondingly. The tvEM approach can provide valuable inference on the extremes of time series, which in the case of infectious disease notifications, allows public health officials to understand the likely scale of outbreaks in the long run.

## Author summary

Dengue is an arbovirus affecting populations across much of the globe. Frequent outbreaks occur, especially in equatorial cities such as Singapore, where the year-round tropical climate, large daily influx of travelers and population density provide the ideal conditions for dengue transmission. Little work has however quantified the peaks of dengue outbreaks, when health systems are likely to be most stretched. Nor have methods been developed to infer differences in exogenous factors which lead to the rise and fall of dengue case counts across extreme and non extreme periods. In this paper, we developed time varying extreme mixture (tvEM) methods to account for the temporal dependence of dengue case counts across extreme and non-extreme periods. tvEM is able to infer differences in climatic forcing across non-extreme and extreme periods of dengue case counts, their temporal dependence as well as estimate suitable thresholds with associated uncertainties to determine dengue case count extremities. Using tvEM, we found no evidence that weather affects dengue case counts in the near term for non extreme periods, but has non-linear and mixed signals in influencing dengue through tvEM parameters in the extreme periods. Using the most appropriate tvEM specification, we found that a high percentile threshold is estimated, with dengue outbreak events far larger than currently observed to be expected in 5, 50 and 75 years. Weather parameters at a 1% scaled increase was found to decrease the long-run expected case counts, but larger increases would lead to a drastic expected rise from the baseline correspondingly. tvEM can provide valuable inference on the extremes of time series, which in the case of infectious disease data, allows public health officials to understand factors and the likely scale of infectious disease outbreaks in the long run.

This is a *PLOS Computational Biology* Methods paper.

## Introduction

An estimated 390 million dengue infections occur annually, imposing major economic and health burdens globally [1]. It is widespread in South-east Asia, with outbreaks occurring annually, sometimes exhibiting synchronous behaviour [2]. Furthermore, it is hyper-endemic within the region due to all four serotypes being in active circulation. Increased urbanization and elevated human movement rates via both domestic and international travel have increased the transmission potential of dengue, particularly across highly connected cities such as Singapore. With favourable vector-breeding conditions due to the year-round tropical climate, a large daily influx of travelers and population density, Singapore has well-suited conditions for dengue infections to occur, with an average of over 100 cases being reported every week from 2000 to 2017.

Primarily, vector control is used to mitigate dengue transmissions in Singapore and its success is evidenced in the decreasing seroprevalence nationally for the past two decades [3–5]. However, this low seroprevalence complicates the implementation of vaccination using the tetravalent Dengvaxia (CYD-TDV) [6, 7] vaccine on the national scale [8] due to potentially longer-term risks of severe dengue in vaccinated but seronegative individuals [9]. Fogging and breeding site reduction, in conjunction with novel biocontrol techniques such as *Wolbachia* are utilized instead to prevent and control dengue epidemics [10]. However, major outbreaks still occur sporadically, with some attributed to population level phenomena such as serotype switching [11–13], in which a large change in the proportion of circulating serotypes lead to an increase in the number of reported infections. While sufficient healthcare capacity is usually available to deal with muted levels of dengue infections, a large and prolonged rise in the number of cases may lead significant impact on public health resources.

Being able to infer the properties of extreme values of dengue transmissions allows public health planning at the national level to be equipped with the information necessary to handle outbreaks. Typically, modelling or forecasting case counts is conducted using mathematical, statistical and machine learning tools [14, 15]. These tools allow understanding of dengue transmission dynamics, aid short to medium term resource planning for disease surveillance and inform decisions about strengthening vector control for outbreak control [16]. However, these tools are used for predicting and inferring case counts to minimize overall error for the entire dataset and are not optimized to characterize extreme conditions such as the peak of dengue outbreaks [17]. Peaks of dengue outbreaks may also not occur in the near term, which may further degrade the quality of predictions for standard models [14, 16]. As a result, it may be difficult to implicitly quantify the long-term risk and scale of these events.

Similar problems exist in other fields such as climatology, oceanography and geography, where the risk and scale of potential calamitous events such as floods, earthquakes and surge storms need to be quantified [18]. Characterization of these rare but serious events are conducted through tools developed from Extreme Value Theory (EVT), with statistical estimation and inference conducted on the extremes of observed events across time and/or space [18]. Some public health work has explored the use of standard EVT tools such as distributional inference on extreme health events such as infectious disease outbreaks; there have been particular applications of EVT to Pneumonia and Influenza (PI) death rates [19] and food-borne disease outbreaks [20], for instance. These works signal its promise for application in a public health setting. Yet, while relatively large leaps have occurred in the development of extreme value theory—such as inference on both extreme and non-extreme portions of data [21], hierarchical modelling on extreme distributions, formulating EVT parameter estimation as a regression problem through Bayesian data augmentation and quantifying space and/or time dependence of extreme values [22, 23]—to the authors’ knowledge, no work in biology nor public health has developed the use of these potentially highly informative extensions of EVT tools.

This paper therefore develops and explores the utility of tools derived from extended extreme value theory to investigate and compare the dynamic signature of extreme and non-extreme periods of dengue transmissions in Singapore. Briefly, we aim to determine thresholds and thus classify extreme and non-extreme periods of dengue transmission, quantify the temporal dependence of extreme and non-extreme periods of dengue transmissions as well as dengue’s potentially non-linear relationship with weather. To do so, we first developed four separate extreme-bulk mixture models which are able to characterize the different dynamic signatures of dengue transmissions, in both extreme and non-extreme periods. These methods were compared against several diagnostic checks, such as residual autocorrelation, quantile-quantile plots, Bayes factor and deviance information criterion, to assess the most suitable model given our data generating process. Next, we explored the potential for climatic fluctuations to affect both extreme and non-extreme periods of dengue transmissions and the differences in transmission behaviour between separate periods. Lastly, we projected the expected scale of dengue events in the long run using the most suitable EVT model and provide discussions on these results.

## Results

### Dengue in Singapore from 2000 to 2017

**Fig 1:**
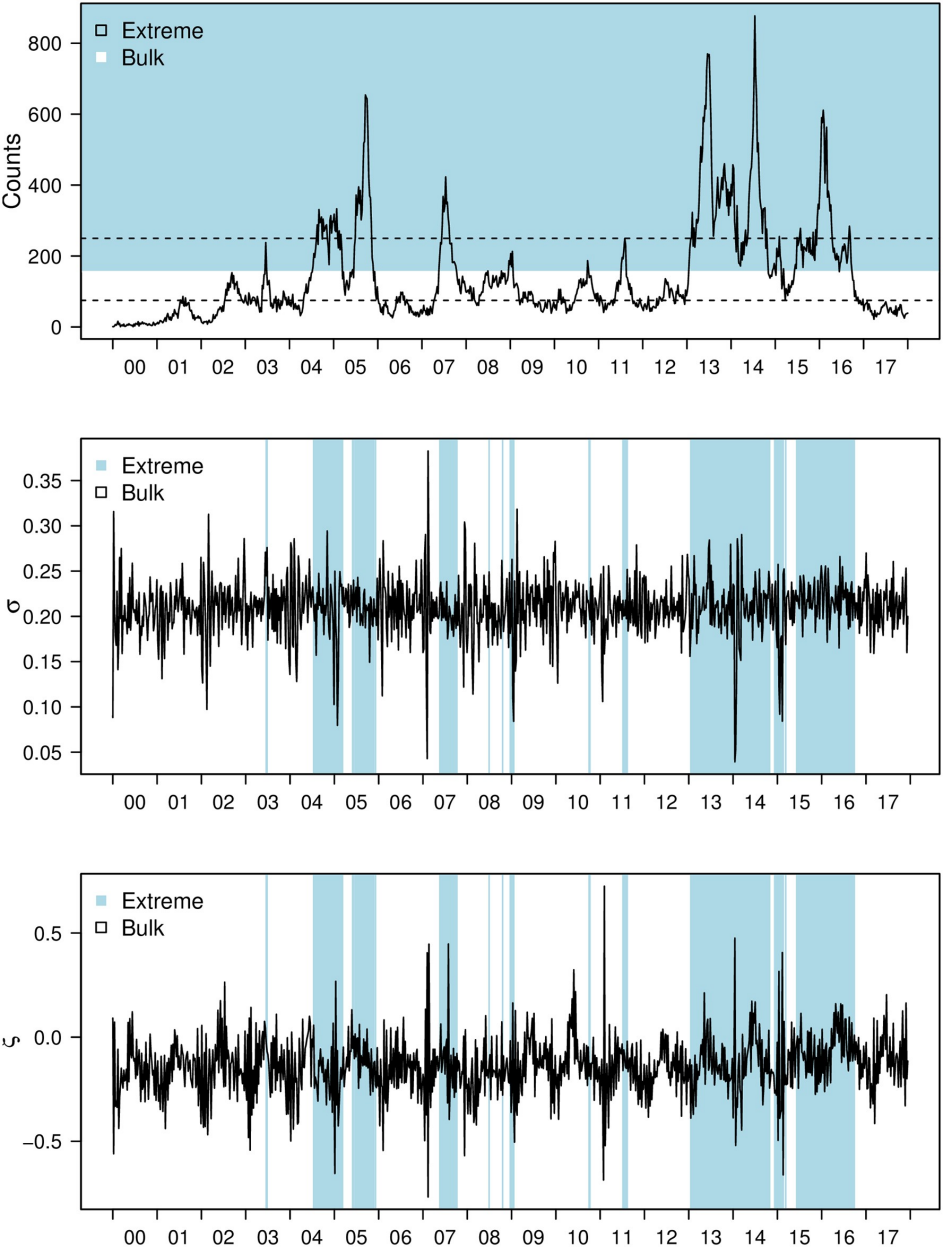
From top to bottom: 1) Dengue case counts from 2000 to 2017 with data above the posterior mean threshold shaded. 95% credible intervals for the threshold are given in dotted lines 2) Posterior mean time-varying scale parameter for constant beta model, with highlighted areas representing timepoints when data are above the threshold from 2000 to 2017 3) Posterior mean time-varying shape parameter for constant beta model, with highlighted areas representing timepoints when data are above the threshold from 2000 to 2017.

An average of over 100 dengue cases are reported every week from 2000 to 2017 in Singapore, with large rises in periods between 2004 to 2005, the middle of 2007, 2013 to 2014 and the end of 2015 and beginning of 2016. On visual inspection of the time series, no clear seasonal pattern is observed, with large rises in dengue observed in 2007 and 2013 to 2016 (Fig 1). We fitted four competing models (M1–M4) to infer the driving patterns of biologically relevant covariates on both elevated (extreme) and baseline (non-extreme) periods of dengue in Singapore from 2000 to 2017.

### Model assessment

Convergence of MCMC chains for parameters across all four competing models was indicated by Gewecke convergence tests, signalling that each marginal parameter posterior space is well explored. Visual inspection of trace plots was also conducted (S1 Fig). Residual autocorrelation for the bulk distribution also indicate that an autoregressive (AR) order 3 model is sufficient to explain dengue transmission dynamics as each lag’s autocorrelation is within the 95% confidence interval bound. Comparing each model’s deviance information criterion (DIC) and log Bayes factor (logBF) showed increasing DIC across models and increasing logBF as models become more complex, indicating that more complex models are penalized as expected from the DIC but overall more favoured in explaining the data according to logBF. In general, a higher logBF and lower DIC indicates a more suitable model for the data. In particular, we obtained the highest DIC and logBF for the model with constant beta term in the tail distribution (Table 1 M4 DIC: 14 268, logBF: 663), followed by a trend of decreasing DIC and increasing logBF in the case where no regression structure was imposed on the tail distribution (Table 1 M3 DIC: 13 111, logBF: 545), no weather variables being added to the bulk distribution (Table 1 M2 DIC: 12 781, logBF: 510), as well as no regression structure being imposed on either bulk nor tail distribution (Table 1 M1 DIC: −1563).

**Table 1:** Model fit for 4 competing mixture models.

Model	DIC	logBF
M1: Gamma + Constant GPD	−1 563	
M2: Normal(AR) + Time-Varying GPD	12 781	510
M3: Normal(AR+weather) + Time-Varying GPD	13 111	545
M4: Normal(AR+weather) + Regression GPD	14 268	663

The quantile-quantile (QQ) plots compare the similarity of fitted distributions to data. Although the DIC for the first model (M1) is low, QQ plots indicate that fitting the gamma distribution to the bulk data leads to significant deviations from the line of unity as compared to regression structures (M2–4): this shows that M1 does not provide distributional characteristics which are similar to the data. For the extreme distribution, QQ plots for the regression structure (M4) generalized Pareto distribution (GPD) has points lying closest to the line of unity, indicating that it is the most suitable structure to account for extreme value data (S1 Fig). There is a high logBF and favourable QQ plots but also higher DIC for M4 compared to other models. However, as regression structure was imposed on both the bulk and tail distribution, the increased penalty on model complexity under DIC is expected. M4 is also further able to compare the effects of biologically relevant covariates across non-extreme and extreme states of dengue transmission dynamics, compared to all other models. Further exposition on results will thus refer to M4 unless specifically mentioned otherwise.

### Inference on extremes of dengue transmissions

**Fig 2:**
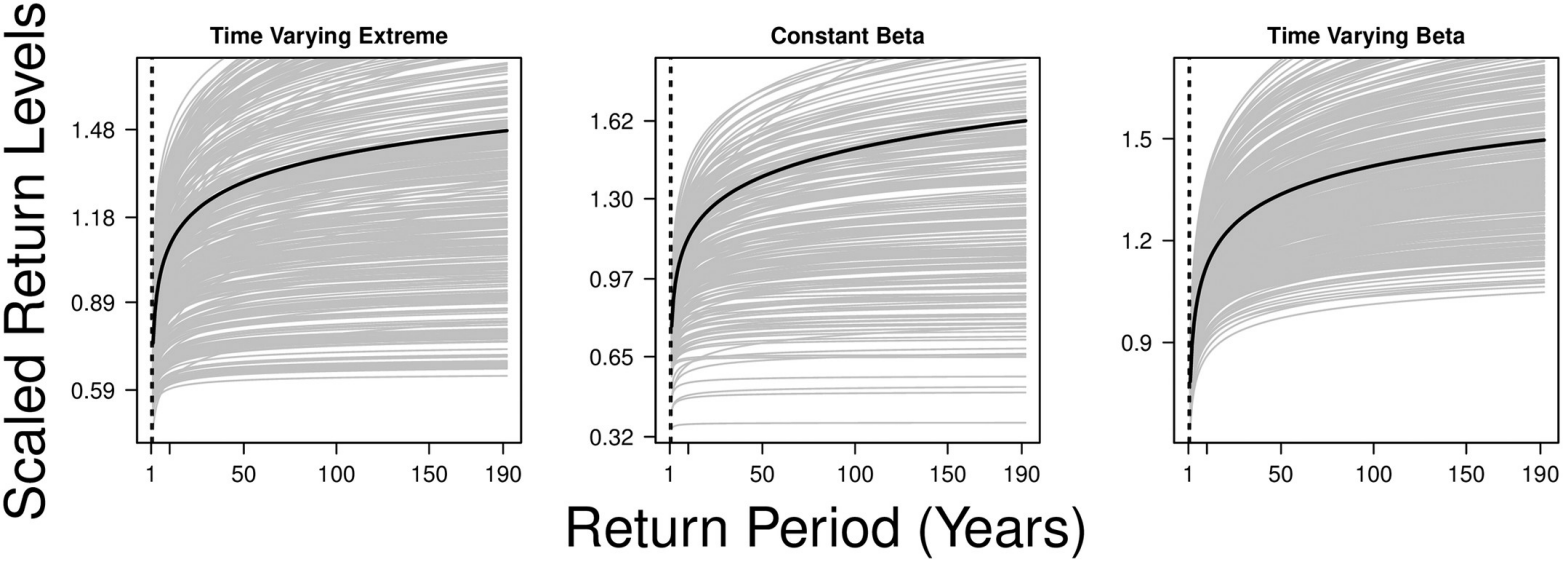
Return levels over a 1 to 190 year return period, with posterior mean estimate given in solid black lines, estimates from the following models are expressed from left to right: 1) Time-varying generalized Pareto distribution 2) Time-varying generalized Pareto distribution with constant beta regression structure 3) Time-varying generalized Pareto distribution with time-varying beta regression structure.

Across 2000 to 2017, when the time varying extreme mixture model (tvEM) was estimated, an observation of around 150 weekly case counts was estimated to be threshold for data to be classified as extreme, with 95% credible interval (CrI) 72 to 244 cases. This corresponds to dengue case counts being above the 70^*th*^ percentile (95% CrI 41.1–83.8) to be taken as extreme (Fig 1a). In periods where weekly dengue case counts are around or above 150, extreme value parameters correspond to larger fluctuations compared to nuisance parameters estimated in non-extreme periods (Fig 1b and 1c). Scaled return level results are consistent across M1–M4 (Fig 2a–2c), with return levels computed by integrating time varying parameters in the tvEM indicate that at baseline levels, an average scaled level of 0.70, 1.30 and 1.45 will be reached in 5, 50 and 75 years, corresponding to an event where 526.6, 1052.2 and 1183.6 weekly dengue case counts are expected to be observed (Fig 2b). These estimated return levels flatten off after 100 years after to a scaled level of around 1.60 (Fig 2b).

### Duality of climatic effects below and above thresholds of dengue transmission

**Fig 3:**
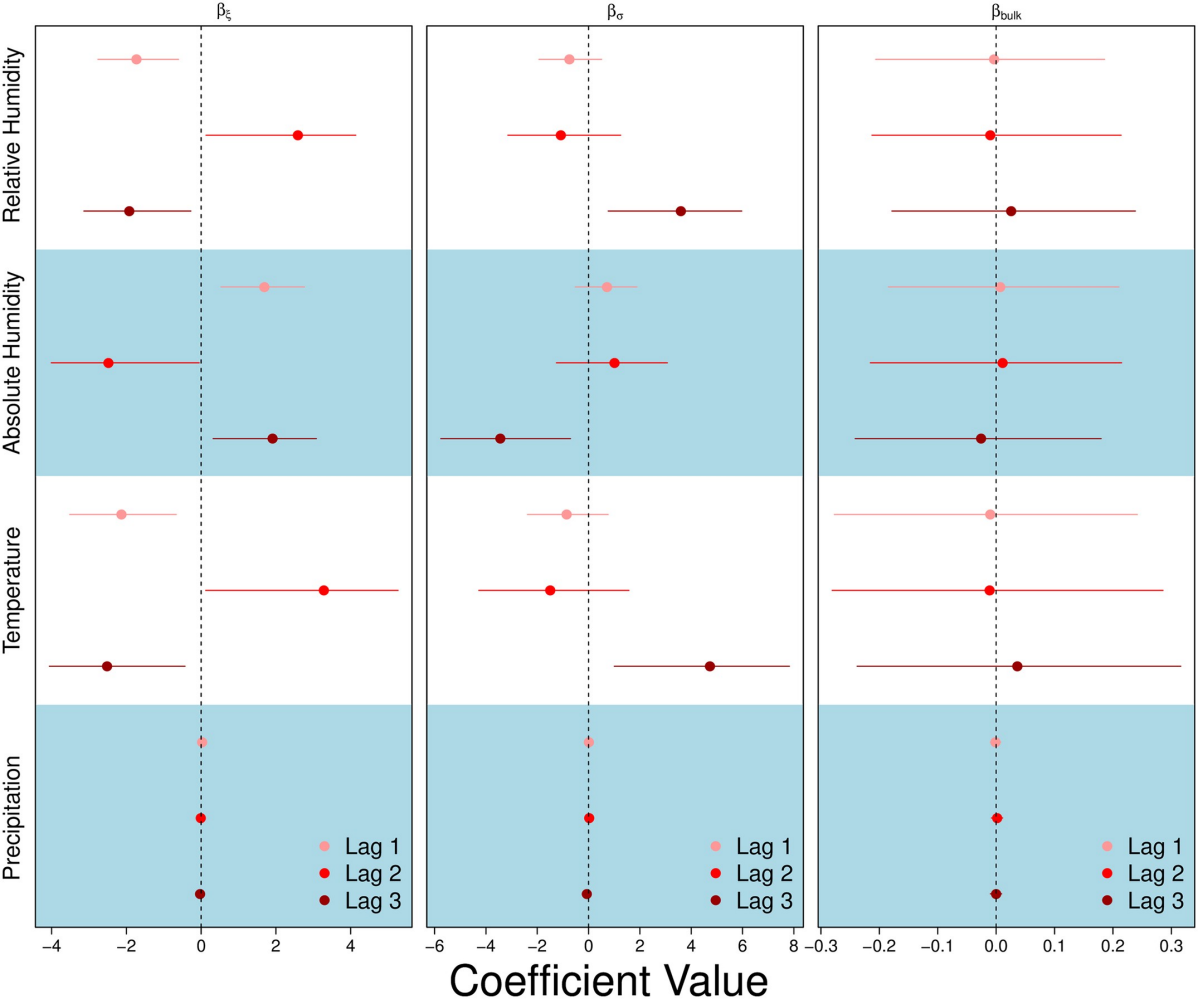
Coefficient plots for the constant beta regression for the generalized Pareto regression parameters and bulk regression. Panels from left to right represent the regression coefficients with the dependent variable being 1) Generalized Pareto distribution shape parameter 2) Generalized Pareto distribution scale parameter 3) Dengue case counts below the threshold.

For the tvEM bulk distribution where the AR model of order 3 was estimated along with weather variables of up to 3 week lags, we found that 95% credible intervals for climatic coefficients all overlap with 0, with posterior mean estimates not lesser or greater than −0.1 and 0.1 respectively. The directions of these coefficients are also mixed, with all 95% credible intervals not crossing more than −0.3 and 0.4. These credible intervals are also consistent in their spread, at around 0.5 to 0.6 in magnitude, asides from precipitation, where smaller intervals are evident and range from −0.01 to 0.011 (Table 2, Fig 3c).

**Table 2:** Coefficient posterior mean and 95% credible intervals for the constant beta regression with the generalized Pareto regression parameters and bulk regression. Columns from left to right represent the regression coefficients with the dependent variable being ^1^Generalized Pareto distribution shape parameter ^2^Generalized Pareto distribution scale parameter ^3^Dengue case counts below the threshold. *Coefficients whose 95% credible intervals are away from 0.

	*β*_*ξ*_		*β*_*σ*_		*β*_bulk_	
Coefficient	Estimate^1^	95% CrI^1^	Estimate^2^	95% CrI^2^	Estimate^3^	95% CrI^3^
Precipitation						
Lag 1	−0.032	(−0.067, 0.003)	-0.069*	(−0.119, −0.016)	0	(−0.01, 0.009)
Lag 2	−0.011	(−0.052, 0.043)	0.017	(−0.05, 0.066)	0.002	(−0.008, 0.011)
Lag 3	0.022	(−0.028, 0.077)	0.01	(−0.053, 0.067)	−0.001	(−0.01, 0.008)
Temperature						
Lag 1	−2.514*	(−4.059, −0.43)	4.724*	(1.005, 7.826)	0.036	(−0.238, 0.316)
Lag.2	3.279*	(0.123, 5.267)	−1.5	(−4.276, 1.572)	−0.011	(−0.281, 0.286)
Lag 3	−2.136*	(−3.513, −0.665)	−0.852	(−2.377, 0.763)	−0.01	(−0.277, 0.242)
Absolute Humidity						
Lag 1	1.912*	(0.32, 3.083)	−3.448*	(−5.759, −0.703)	−0.026	(−0.241, 0.18)
Lag 2	−2.479*	(−4.01, −0.051)	1.014	(−1.253, 3.071)	0.011	(−0.215, 0.215)
Lag 3	1.693*	(0.531, 2.764)	0.717	(−0.518, 1.881)	0.007	(−0.184, 0.21)
Relative Humidity						
Lag 1	−1.924*	(−3.136, −0.275)	3.597*	(0.765, 5.969)	0.026	(−0.178, 0.238)
Lag 2	2.587*	(0.133, 4.135)	−1.077	(−3.143, 1.249)	−0.01	(−0.212, 0.214)
Lag 3	−1.729*	(−2.761, −0.604)	−0.746	(−1.933, 0.51)	−0.004	(-0.206, 0.186)

However, we found that 95% credible intervals for relative humidity, absolute humidity and temperature coefficients exclude 0 across certain lags in the tvEM extreme distribution. Specifically for the weather coefficients governing the extreme distribution scale parameter *β*_*σ*_, we found negative effects coming from precipitation (Table 2: −0.069, 95% CrI: −0.119, −0.016) and absolute humidity (Table 2: −3.448, 95% CrI: −5.759, −0.703) at a 1 week lag. Correspondingly, positive effects from temperature (Table 2: 4.724, 95% CrI: 1.005, 7.826) and relative humidity (Table 2: 3.597, 95% CrI: 0.765, 5.969) at a 1 week lag also. The magnitude of the precipitation coefficient and the spread of the 95% credible intervals are similarly smaller than that of other weather coefficients. For weather coefficients governing the extreme distribution scale parameter, all 2 to 3 week lag variables have 95% credible intervals containing 0 (Table 2).

For the weather coefficients governing the extreme distribution shape parameter *β*_*ξ*_, the precipitation coefficients had all 95% credible intervals containing 0 from 1 to 3 weeks lag. Conversely, all other weather covariates had 95% credible intervals for their coefficients that excluded 0 for 1 to 3 weeks’ lag term. These coefficients also vary in direction across lags for the same variable. For example, the temperature coefficient had 1 and 3 weeks lag being negative in sign, but the 2 weeks lag was positive (Table 2). For the 1 week lag climate coefficients, the coefficients governing the extreme distribution shape parameter *β*_*ξ*_ and the extreme distribution scale parameter *β*_*σ*_ are in opposite directions always (Table 2, Fig 3). The absolute humidity coefficient for the extreme distribution shape parameter is positive but the same coefficient for the extreme distribution scale parameter is negative. Conversely, the relative humidity coefficient for the extreme distribution shape parameter is negative but the same coefficient for the extreme distribution scale parameter is positive (Table 2).

### Nonlinearities in climatic effects on extremes of dengue transmission

We have lastly examined how weather coefficients governing tvEM extreme distributions affect expected return levels. We have only included coefficients whose 95% credible intervals excluded 0, namely, temperature, absolute humidity and relative humidity. In general, we found the effects of weather on return levels to be highly nonlinear and dependent on the specific value of shocks applied to each coefficients.

**Fig 4:**
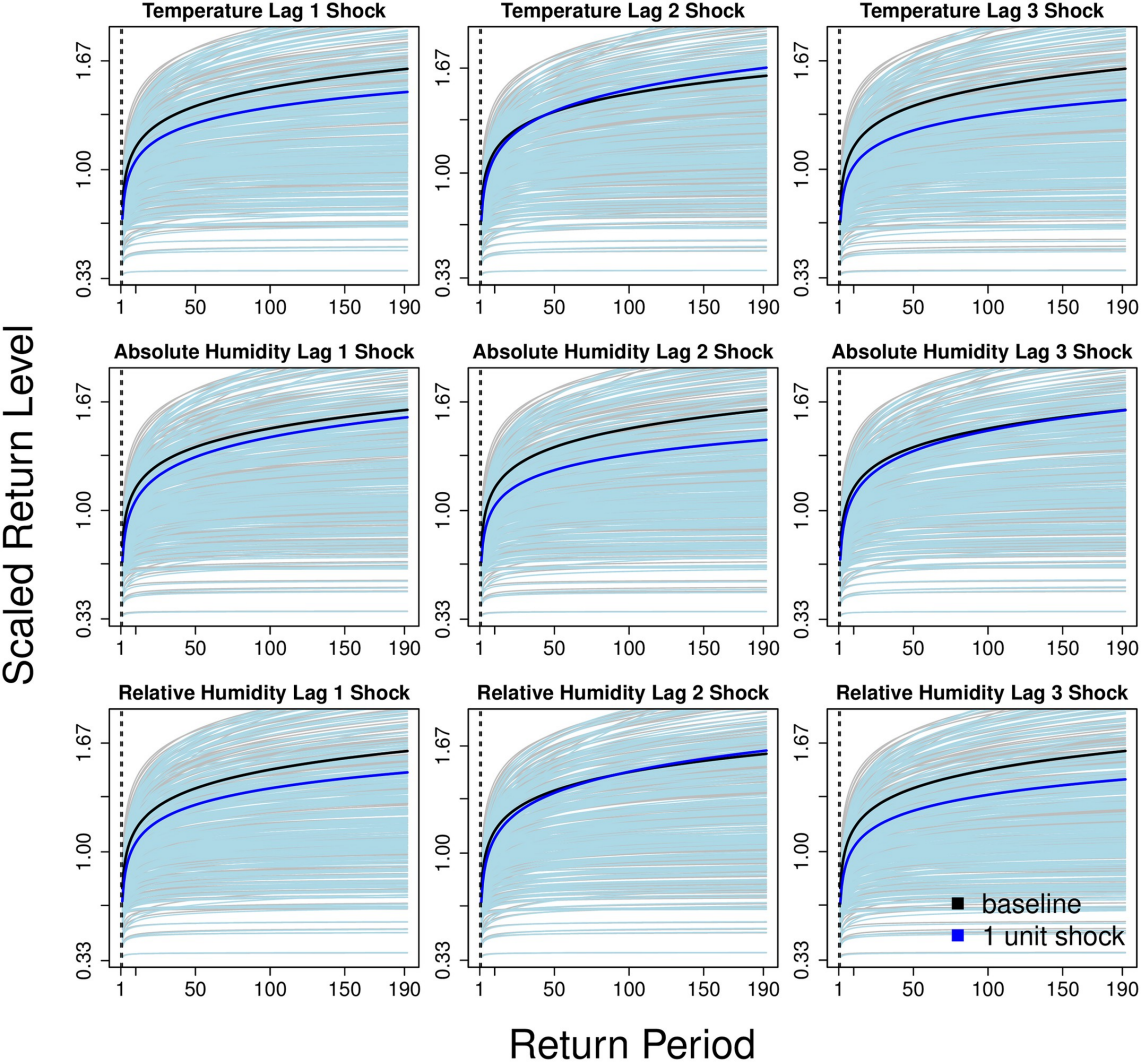
Posterior mean return levels at baseline (black solid line) and posterior mean return levels (blue solid line) given a 1 unit shock on each respective climatic parameter for the constant beta regression over 1 to 190 years.

For a 1% positive shock across each parameter at each lag, we found in general a decrease in the expected return levels across time for temperature lag 1 and 3, absolute humidity lag 1 and 2 and relative humidity at lag 1 and 3 (Fig 4). However, the changes in return levels are almost negligible when shocks are applied to temperature lag 2, absolute humidity lag 3 and relative humidity lag 2, corresponding roughly to points when the effects of weather on the scale and shape parameter are positive and negative respectively for temperature lag 2 and absolute humidity lag 3. They also correspond to instances when the effects of weather on the scale and shape parameter are negative and positive, respectively, for relative humidity lag 3 (Fig 3a–3c).

**Fig 5:**
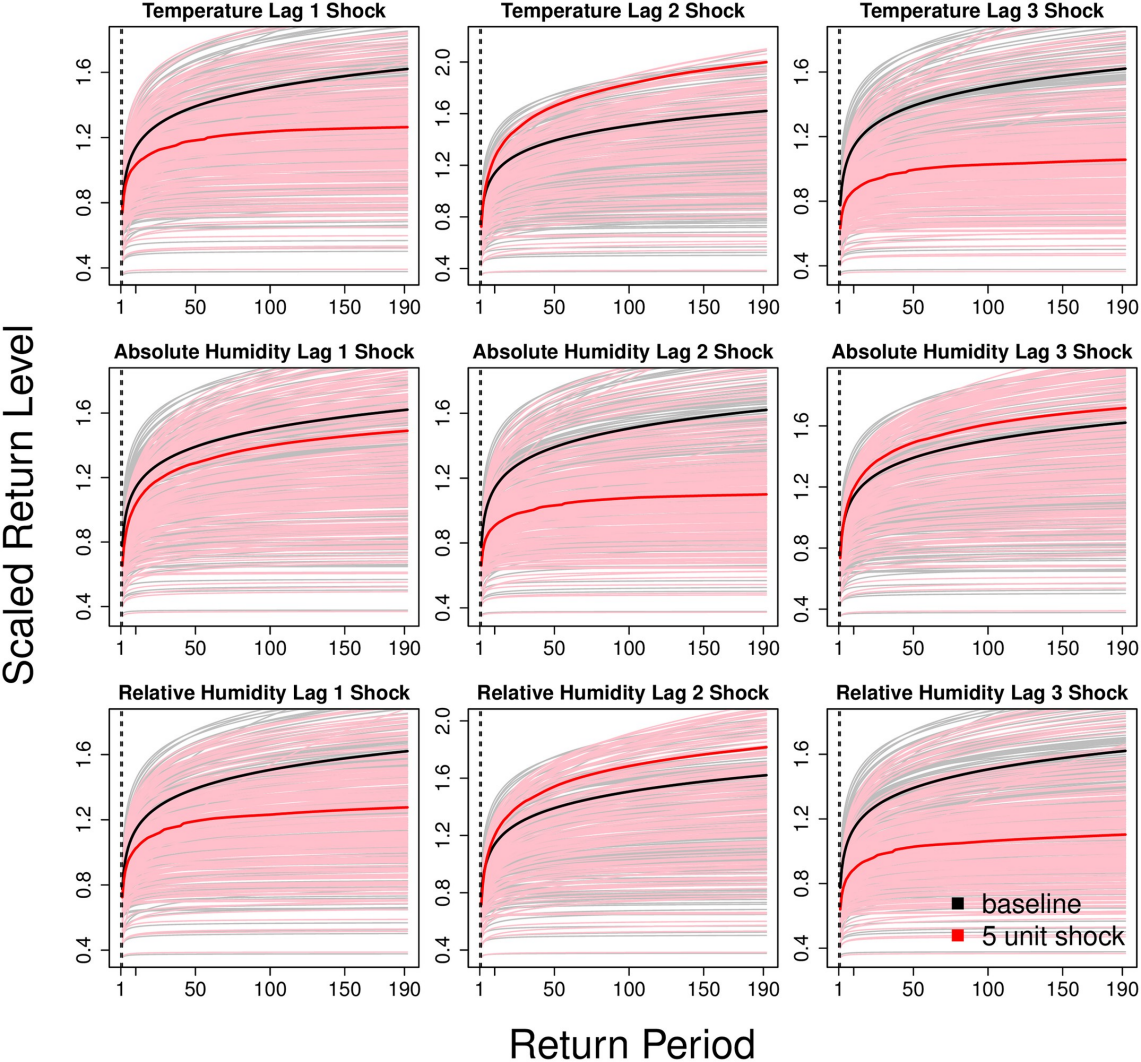
Posterior mean return levels at baseline (black solid line) and posterior mean return levels (red solid line) given a 5 unit shock on each climatic parameter for the constant beta regression over 1 to 190 years.

For a 5% independent, positive shock across each parameter at each lag, we found markedly larger magnitudes of change in the expected return levels across time (Fig 5). The same decreases were found in the expected return levels across time for temperature lag 1 and 3 and relative humidity lag 1 and 3, but these are markedly larger compared to the 1% shock (Figs 4 and 5). However, with a 5% shock in absolute humidity lag 1 and 2, their effects on return levels are now positive. The formerly negligible change when shocks are applied to temperature lag 2, absolute humidity lag 3 and relative humidity lag 2 are now positive and rise much more steeply after extending the return period beyond 10 years (Fig 5).

## Discussion

Results indicate that the tvEM model with regression structure on the extreme and non-extreme distributions of dengue case counts (M4) could characterize the data well under model assessment checks (Table 1, S1 Fig). Using M4 to infer weather effects below and above thresholds of dengue transmission showed a duality of weather effects below and above thresholds of dengue transmission, where it is found to affect the GPD distribution parameters but not case counts below the threshold (Table 2). The effects of weather on extremes in dengue transmission are also highly nonlinear and severe, with a mild to moderate increase in weather observations leading to long run changes in expected return levels over time (Figs 4 and 5).

Inference of extreme and non-extreme periods of dengue transmissions across time can be conducted using tvEM, with the model being able to characterize the temporal dependence of dengue case counts across time. In general, inference through tvEM indicates that weather measurements such as absolute humidity, relative humidity and temperature are able to influence projected return levels over time, but these same variables do not have a near term influence on dengue case counts directly. These results are in line with previous work [24] which found that dengue case counts in Singapore can be sufficiently described autoregressively. While said weather variables may exert influence on dengue case data over longer timelags, spurious correlations may result from such an analysis due to temporal dynamics already adequately accounted for in the bulk distribution.

Our results found that a mild to moderate shock in weather variables would lead to considerable changes in the expected return levels over a long return period. As indicated from this analysis, independent 1% positive shocks on weather variables on 1 to 3 week lags would lead to either little change or decreases in projected return levels, while larger 5% shocks would more often lead to increases in projected return levels in dengue case counts across time (Figs 4 and 5). Other work has also revealed non-linear, interactive effects of weather on dengue transmission dynamics, wherein weather measurements may often exhibit mixed, concave and/or second order interaction effects on reported cases. For example, large rises in precipitation may reduce viable breeding grounds immediately, but may also lead to the formation of future breeding grounds through rainwater accumulation [25–29]. Evidence also suggests that mosquito ovipositing, development from mosquito larva to adult, biting rate and viral incubation time in the dengue vector are enhanced at raised ambient temperatures, but these effects have marginal gains at much higher temperatures [30, 31]. Similarly, optimally humid conditions can boost vector egg development and subsequent adult population size that may itself be correlated with the transmission potential of dengue [32–34].

Using tvEM, events of at least 526.6, 1052.2 and 1183.6 weekly dengue case counts are expected to occur in 5, 50 and 75 years respectively in Singapore. This notion of return levels is helpful for public health resource planning purposes. In the case of dengue, the expected surge in vector control, primary and secondary care required to deal with dengue fever (DF) and dengue hemorrhagic fever (DHF) can easily be computed using prior data on DF/DHF occurrence [35] and the expected number of visits and medication required for DF/DHF patients [36], both as a factor of the return levels. This method is also easily translatable to other diseases in different localities, regions or countries where disease case counts are collected over a long period of time, where the notion of exceeding a certain number of cases in any disease can be obtained for risk management purposes.

The methods described in this paper are also able to resolve certain issues in empirical EVT applications. Thresholds are estimated automatically using the MCMC approach employed in this paper, with threshold estimation uncertainty also quantified in its posterior distribution. This allows individuals to ignore the often subjective methods to select thresholds using graphical diagnostic tests and various rules of thumb approaches while working with extreme values empirically [17, 18, 37, 38]. Next, imposing an hierarchical temporal structure in tvEM also resolves the temporal dependence of observed data for the extreme value distribution, allowing stronger inference on time dependent data such as dengue case counts. This could easily be translated for use in other endemic infectious disease data such as influenza, for which case counts are also temporally dependent. Lastly, by inducing a regression structure on the extreme value distribution, statistical learning on relevant covariates could be conducted, which then allows meaningful interpretations of the covariates on projected return levels. These covariate effects could then also be compared to that of non-extreme value data within a single model structure, as evidenced by tvEM in this paper.

Several limitations are recognised. First, explicitly quantifying the effect of covariates is difficult in the extreme portion of data, as their effects are on the time-varying extreme value distribution parameters rather than the dependent variable itself. However, while direct comparison between extreme and non-extreme portions of the data is challenging, we used the shock notion to explore the effects on return levels. Time varying parameters estimated over the extreme value distribution are also difficult to interpret and return levels have to be computed after integrating out the parameters sampled over the range of values over the threshold. The large number of parameters estimated due to time varying extreme value parameters also makes the MCMC estimation approach slow and future work should explore the use of approximate but faster estimation methodologies such as particle MCMC or approximate Bayesian computation [39, 40]. The suitability for each EVT tool on data also cannot be evaluated directly using quantities such as the mean-square error or *R*^2^ as estimation above the threshold provides a time varying regression on parameters but not observed case counts unlike the bulk distribution formulation, leading us to use other diagnostic measures such as DIC or logBF in model comparison. The results of this paper are also only based on passive surveillance data on dengue case counts in Singapore, and may not apply to other health systems with different case definitions or surveillance systems. Lastly, while dengue case counts are plausibly spatio-temporally dependent and may have a large number of past case counts, different multicollinear weather lags affecting contemporaneously reported dengue cases, only the most parsimonious model adequately accounting for temporal dynamics is used due to the inherent difficulty in estimating a time varying distribution with regression structure.

Future work can explore nesting sparse regression priors or spatio-temporal priors on the extreme value regression to infer and incorporate spatio-temporal behaviours or the effect of many covariates on dengue extremes. Incorporation of sparse regression priors may also resolve the issue of multicollinearity between many variables in both bulk and extreme portions of data [41–43]. When data become available, the same assessment checks described in this paper can also be conducted on separate dengue time series to examine model validity, climate-extreme value exposure-response relationships and disparities in return levels.

The methods developed in this paper are easily applicable to any other infectious disease where time series are recorded over a sufficient period of time. These methods are able to conduct statistical learning of covariates on extreme and non-extreme periods of time series data. Lastly, tvEM is able to provide meaningful notions of return levels on a pre-specified return period, which in the case of infectious disease data, allows public health officials to prepare for the likely scale of an infectious disease outbreak in the long run.

## Methods

### Data

Dengue incidence data in Singapore are collected by the Ministry of Health with mandatory notification of virologically confirmed or laboratory-confirmed cases. Laboratory confirmation of dengue cases is conducted through (1) nonstructural protein 1 (NS1) antigen detection, viral RNA detection by polymerase chain reaction (PCR), or (2) immunoglobulin M detection [44, 45]. Data are publicly available from the Infectious Disease Bulletin, published weekly by the Ministry of Health, Singapore. Data is available from 2010 to 2017. No ethical approval is required for this study.

Weather data were obtained from ERA5, published by the European Centre for Medium-Range Weather Forecasts. ERA5 provides hourly estimates across a 30km grid [46], which we aggregate nationally over a weekly timescale and spatially averaged for Singapore. Mean, minimum and maximum air temperature at 2m is calculated to represent thermal forcing and stress on vector population growth, and weekly total rainfall is obtained for breeding site availability. Air temperature and dewpoint temperature is utilized to calculate saturation vapor pressure and actual vapor pressure using Teten’s formula, with which relative and absolute humidity could then be estimated using standard formula [47]. Data are available from 2010 to 2017.

### Extreme value mixtures to infer bulk and extreme periods of dengue

We have used mixtures of bulk and extreme distributions as our key framework to derive statistical inference on both non-extreme and extreme periods of dengue transmission dynamics. The general structure of our model follows (1), where *y*_*t*_ denotes our data at timepoint *t*, *F*_*t*_(*y*_1:*t*_|Θ) the cumulative distribution function of our model, *H*(*y*_*t*_; *u*, Θ_−*u*_) the bulk cumulative distribution with parameters Θ_−*u*_ below the threshold *u* and *G*(*y*_*t*_|*u*, Θ_*u*_) the tail cumulative distribution with parameters Θ_−*u*_ above the threshold *u*.

$$\begin{array}{c} F_{t}\left(y_{1:t}|\Theta \right)=\{\begin{array}{cc} H(y_{t};u,\Theta_{-u}) & \text{if}\;y_{t}<u\\ H\left(u;\Theta_{-u}\right)+\left(1-H\left(u;\Theta_{-u}\right)\right)G\left(y_{t};u,\Theta_{u}\right) & \text{if}\;y_{t}\geq u \end{array} \end{array}$$ (1)

#### Bulk distribution

Four competing models were estimated, first, the canonical case where no regression structure was imposed on *y*_*t*_, with *h*(*y*_*t*_; *u*, Θ_−*u*_) ∼ Gamma(*a*, *b*) and *g*(*y*_*t*_|*u*, Θ_*u*_) ∼ GPD(*u*, *ξ*, *σ*), where GPD refers to the generalized Pareto distribution [23, 48]. Next, the autoregressive (AR) structure without (M2) or with (M3) weather variables (2) was imposed to the bulk distribution with normally distributed errors. Namely, *P* denotes the AR order, with *J* being the number of exogenous variables and *K* the maximal number of lags estimated for exogenous variables. *X*_*t*−*k*,*j*_ refers to the exogenous variable at lag *k* of type *j*. AR and lagged parameters to be estimated are Θ_−*u*_ = {*β*, *α*, *σ*_*b*_}. *ϵ* is some noise term with 0 mean and second moment $\sigma_{b}^{2}$. The AR structure was used for easily interpretable parameters and being able to capture the time-dependent behaviour of dengue case data. It is also supported by a large body of work using AR as a baseline to both understand and forecast dengue transmissions [34, 49–51].

$$\begin{array}{c} y_{t}=\beta_{0}+\sum_{i=1}^{P}y_{t-i}\beta_{i}+\sum_{j=1}^{J}\sum_{k=1}^{K}X_{t-k,j}\alpha_{k,j}+\epsilon \end{array}$$ (2)

$$\epsilon ∼N(0,\sigma_{b}^{2})$$

#### Tail distribution

For values above some threshold *u*, it is standard to fit data using the generalized Pareto distribution (GPD) with Θ_*u*_ = {*ξ*, *σ*, *u*} shape, scale and location parameters to be estimated respectively. Our first model (M1) considers the canonical, static distribution with no regression structure imposed on the GPD (3).

$$\begin{array}{c} G\left(y_{t};u,\Theta_{u}\right)=\{\begin{array}{cc} 1-(1+\frac{\xi (y_{t}-u)}{\sigma }{)}^{-1/\xi }) & \text{if}\;\xi \neq 0\\ 1-\text{exp}(-\left(y_{t}-u\right)/\sigma ) & \text{if}\;\xi =0 \end{array} \end{array}$$ (3)

We then consider imposing time varying parameters on the GPD, with a fixed threshold *u*, but having the scale and shape parameter vary across time Θ_*u*,*t*_ = {*ξ*_*t*_, *σ*_*t*_, *u*} (4). The additional flexibility of using a time varying structure was imposed on *ξ*_*t*_, *σ*_*t*_ as observed dengue case counts may fluctuate greatly over time, especially in dengue hyper-endemic Singapore, which can skew parameter estimation if we only consider static parameters for the GPD distribution. Furthermore, imposing a suitable structure for time variation allows incorporation of dengue case count autocorrelations from successive timepoints. Lastly, past information on model parameters can be incorporated contemporaneously, strengthening inference procedures for the GPD.

$$\begin{array}{c} \Theta_{u,t}=G\left(\Theta_{u,t-1},\Theta_{u,t-2},\ldots ,\Theta_{u,t-g},w_{t}\right) \end{array}$$ (4)

#### Temporal dependence

The time evolution of our GPD parameters follow some function *G* with input variables being the lagged GPD parameters and *w*_*t*_ some noise term (4). The time varying GPD parameters were considered using two functional forms. Firstly, we consider the simplest case of a first order dynamic linear model [52], where parameters follow random walk state equations, with some white noise terms *w*_*ξ*,*t*_ and *w*_*σ*,*t*_ for the shape and scale equations (M2-3) given some initial information drawn from *θ*_*ξ*,0_ ∼ *N*(*m*_*ξ*,0_, *C*_*ξ*,0_) and *θ*_*σ*,0_ ∼ *N*(*m*_*σ*,0_, *C*_*σ*,0_). We log-transform *ξ*_*t*_, *σ*_*t*_ to allow parameters to be within the allowable bounds for the GPD (*ξ* > −1) with *lξ*_*t*_ = log(*ξ*_*t*_ + 1) and *lσ*_*t*_ = log(*σ*_*t*_):

$$\begin{array}{c} l\xi_{t}=\theta_{\xi ,t}+v_{\xi ,t}\;v_{\xi ,t}∼N\left(0,1/V_{\xi }\right)\;\theta_{\xi ,t}=\theta_{\xi ,t-1}+w_{\xi ,t}\;w_{\xi ,t}∼N\left(0,1/W_{\xi }\right) \end{array}$$

$$\begin{array}{c} l\sigma_{t}=\theta_{\sigma ,t}+v_{\sigma ,t}\;v_{\sigma ,t}∼N\left(0,1/V_{\sigma }\right)\;\theta_{\sigma ,t}=\theta_{\sigma ,t-1}+w_{\sigma ,t}\;w_{\sigma ,t}∼N\left(0,1/W_{\sigma }\right) \end{array}$$

Secondly, to allow inference and comparison between the possible effects that weather has on non-extreme and extreme periods of dengue transmissions, we imposed lagged regression structures on the time-varying GPD parameter (M4). To belabour, we allowed inference on *lξ*_*t*_, *lσ*_*t*_ by additionally imposing state-determining Eqs (5) and (6). In M3, *lξ*_*t*_, *lσ*_*t*_ is influenced by the effect of *X*_*t*−*k*,*j*_ weather variables with a maximum of *J* weather terms and *K* lags. The parameters *β*_*ξ*,*j*,*k*_, *β*_*σ*,*j*,*k*_ determine the degree of influence that weather variables have on the GPD parameters.

$$\begin{array}{c} l\xi_{t}=\beta_{\xi ,0}+\sum_{j=1}^{J}\sum_{k=1}^{K}\beta_{\xi ,j,k}X_{t-k,j}+v_{\xi ,t}\;v_{\xi ,t}∼N\left(0,1/V_{\xi }\right) \end{array}$$ (5)

$$\begin{array}{c} l\sigma_{t}=\beta_{\sigma ,0}+\sum_{j=1}^{J}\sum_{k=1}^{K}\beta_{\sigma ,j,k}X_{t-k,j}+v_{\sigma ,t}\;v_{\sigma ,t}∼N\left(0,1/V_{\sigma }\right) \end{array}$$ (6)

### Model estimation

We estimated all models (M1–M4) using Markov chain Monte Carlo (MCMC) approaches. In general, Gibbs sampling was conducted when suitable conditional conjugate distributions can be derived, such as the case of regression parameters in the bulk distribution or their respective noise terms. For the constant and time varying GPD parameters, there are no known conditionally conjugate priors. Marginal sampling of their parameters was conducted using Metropolis or Metropolis-Hastings steps instead. For all models, a total of 10 000 MCMC steps were taken with a burnin of 1000.

Briefly, for the first model where parameters of interest are the gamma bulk shape and rate parameters *a*, *b* and static GPD tail distribution *ξ*, *σ*, *u*, we conducted Metropolis-within-Gibbs steps for each of their respective marginal distributions with proposal distributions tuned to allow efficient exploration of the posterior space. This was also conducted due to the GPD distribution not having any known conjugate prior distributions, which precludes the use of more efficient Gibbs sampling. We sampled *a*, *b*, *ξ*, *u* using truncated normal distributions and *σ* using either a wide Gamma or truncated normal distribution depending on the sign of *ξ*. For time varying tail distribution, random walk Metropolis steps were taken for ${\left\{l\xi_{t}\right\}}_{t=1}^{T}$ and ${\left\{l\sigma_{t}\right\}}_{t=1}^{T}$ with the proposal distribution a symmetric normal distributions with mean given by the previous iteration. With appropriate prior choices on the regression terms and second moments {*V*_*ξ*_, *W*_*ξ*_, *θ*_*ξ*,*t*_, *V*_*σ*_, *W*_*σ*_, *θ*_*σ*,*t*_, *β*, $\sigma_{b}^{2}$, *u*}, where diffuse normal priors are placed for regression parameters and inverse-gamma priors for their respective second moment, conjugate conditional posterior distributions of these terms allow for Gibbs sampling. Full technical details are explicitly provided in the supplementary information.

### Model assessment

Convergence of MCMC chains was first assessed by visual inspection of trace plots and Gewecke convergence diagnostic checks. Residual autocorrelation is computed for up to 20 week lags to ensure that the transmission dynamics of dengue are properly accounted for and to determine the maximum autoregressive lag order for each specification. Quantile-Quantile plots are used to see whether the bulk and tail specifications adequately account for the data structure. The log Bayes factor was computed using naïve Monte Carlo simulation and deviance information criterion as detailed in S1 Text to provide summary measures of model fit and model appropriateness to data.

### Model inference

As we have found the constant beta model (M4) to be favourable on model assessment, we define only return levels for that particular model for clear exposition. Estimation of the GPD provides us with return levels ${\hat{z}}_{r}$ for the *r*-year. This is the level expected to be exceeded once every *r* years, with $\hat{\lambda_{u}}$ the empirical threshold exceedance rate observed in the data set. We generalize return levels from the static distribution case to account for the presence of time varying GPD parameters which are also affected by other covariates in M3 (7).

$$\begin{array}{c} {\hat{z}}_{r,t}(\hat{u},{\hat{\sigma }}_{t},{\hat{\xi }}_{t},{\hat{\beta }}_{\xi },{\hat{\beta }}_{\sigma },X)=\{\begin{array}{c} \hat{u}+\frac{{\hat{\sigma }}_{t}\left({\hat{\beta }}_{\sigma },X\right)}{{\hat{\xi }}_{t}\left({\hat{\beta }}_{\xi },,X\right)}({\left(r\hat{\lambda_{u}}\right)}^{{\hat{\xi }}_{t}\left({\hat{\beta }}_{\xi },X\right)}-1),\;\text{if}\;{\hat{\xi }}_{t}\left({\hat{\beta }}_{\xi },,X\right)\neq 0\\ \hat{u}+{\hat{\sigma }}_{t}\left({\hat{\beta }}_{\sigma },X\right)\text{log}\left(r\hat{\lambda_{u}}\right),\;\text{if}\;{\hat{\xi }}_{t}\left({\hat{\beta }}_{\xi },,X\right)=0 \end{array} \end{array}$$ (7)

The expression (7) above provides some return level for the *r*-year at every time-point *t* where GPD parameters were estimated, given some specified return period *r*. Integrating out the expression across time provides us with the mean return level for dengue transmissions over the tail dataset (8).

$$\begin{array}{c} E_{t}\left[{\hat{z}}_{r,t}\right]=\int {\hat{z}}_{r,t}(\hat{u},{\hat{\sigma }}_{t},{\hat{\xi }}_{t},{\hat{\beta }}_{\xi },{\hat{\beta }}_{\sigma },X)\;dt \end{array}$$ (8)

The subsequent effect of some weather variable on expected return levels can be inferred by comparing the return levels at baseline versus some unit shock *ω*_*l*,*p*_ for some prespecified variable and lag across the dataset (9) and (10). It was then back transformed to allow computation of return levels in (7) and (8) given the new scenario. In this paper, we have looked at the effect of 1% and 5% increases in the observed range of each of our weather variables on our return levels.

$$\begin{array}{c} {\hat{l\xi }}_{t,shock}=\sum_{j\neq l}^{J}\sum_{k\neq p}^{K}{\hat{\beta }}_{\xi ,j,k}X_{t-j,k}+{\hat{\beta }}_{\xi ,l,p}\left(X_{t-l,p}+\omega_{l,p}\right) \end{array}$$ (9)

$$\begin{array}{c} {\hat{l\sigma }}_{t,shock}=\sum_{j\neq l}^{J}\sum_{k\neq p}^{K}{\hat{\beta }}_{\sigma ,j,k}X_{t-j,k}+{\hat{\beta }}_{\sigma ,l,p}\left(X_{t-l,p}+\omega_{l,p}\right) \end{array}$$ (10)

## Supporting information

S1 Text Details of MCMC procedures and model assessment checks. (PDF)

S1 Fig Results of model asssessment checks. (PDF)
