# Supplementary material for: Time varying methods to infer extremes in dengue transmission dynamics
Source: PLoS Comput Biol. 2020 Oct 12;16(10):e1008279. doi: 10.1371/journal.pcbi.1008279 (PMC7595636; doi:10.1371/journal.pcbi.1008279)
Supplement: S1 Text — (PDF) [file pcbi.1008279.s001.pdf]

Appendix 1

September 24, 2020

Contents

1 Mode1 1: Gamma bulk distribution with constant generalized pareto distribution 1

1.1 Sampling ξ . . . 2
1.2 Sampling σ . . . 2
1.3 Sampling u . . . 2
1.4 Sampling α and β . . . 2

2 Model 2 & 3: Constant bulk regression with time-varying generalized pareto distribution 2

2.1 Sampling $\boldsymbol{\beta}$ and σ^2 . . . 3
2.2 Sampling $\{l\xi_t\}_{t=1}^T$ . . . 3
2.3 Sampling $\{l\sigma_t\}_{t=1}^T$ . . . 3
2.4 Sampling u . . . 4

3 Model 4: Constant bulk regression and generalized pareto distribution regression 4

3.1 Sampling β and σ^2 . . . 4
3.2 Sampling $\{l\xi_t\}_{t=1}^T$ . . . 4
3.3 Sampling $\{l\sigma_t\}_{t=1}^T$ . . . 5
3.4 Sampling u . . . 5

4 Model 5: Constant bulk regression and time-varying generalized pareto regression 5

4.1 Sampling β and σ^2 . . . 5
4.2 Sampling $\{l\xi_t\}_{t=1}^T$ . . . 5
4.3 Sampling $\{l\sigma_t\}_{t=1}^T$ . . . 6
4.4 Sampling u . . . 6

5 Model Assessment 6

1 Mode1 1: Gamma bulk distribution with constant generalized pareto distribution

Below the threshold u, the bulk distribution is a gamma distribution, with shape and scale parameters α and β. Above the threshold u, we place the generalized pareto distribution (GPD) with parameters (ξ, σ, u):

$$G(y; u, \Theta_u) = \begin{cases} 1 - \left(1 + \frac{\xi_(y-u)}{\sigma})^{-1/\xi}\right) & \text{if } \xi \neq 0 \\ 1 - \exp(-(y-u)/\sigma) & \text{if } \xi = 0 \end{cases} \quad (1)$$

We apply a non-informative prior for (ξ and σ) following Castellanos and Cabras 2007 and Do Nascimento 2012:

$$p(\xi, \sigma) \propto \sigma^{-1}(1+\xi)^{-1}(1+2\xi)^{-\frac{1}{2}}$$

A truncated normal distribution with parameters (μ_u, σ_u^2) with the following density is placed on u:

$$p(u|\mu_u, \sigma_u^2) \propto \frac{1}{\sqrt{2\pi\sigma_u^2}} \frac{exp\{-0.5(u-\mu_u)^2/\sigma_u^2\}}{\Phi(\mu_u/\sigma_u)}$$

With the following priors set for parameters below the threshold,

$$\alpha \sim Ga(a, b)$$

$$\mu \sim Ga(c, d)$$

where $\mu = \frac{\alpha}{\beta}$

1.1 Sampling ξ

ξ^* is sampled from a $N(\xi^{(s)}, V_\xi)I(-\sigma^{(s)}/(M-u^{(s)}, \infty)$ distribution, where M $= max(x_1, ..., x_n)$. Therefore $\xi^{(s+1)} = \xi^*$ is drawn with acceptance probability α_ξ:

$$\alpha_\xi = \min\left(1, \frac{p(\theta^*|x)\Phi((\xi^{(s)} + \sigma^{(s)}/(M-u^{(s)}))/\sqrt{V_\xi})}{p(\tilde{\theta}|x)\Phi((\xi^* + \sigma^{(s)}/(M-u^{(s)}))/\sqrt{V_\xi})}\right)$$

1.2 Sampling σ

If $\xi^{(s+1)} \geq 0$ then σ^* is sampled from a $Ga(a_s, b_s)$ distribution with $a_j = (\sigma^{(s)})^2/V_\sigma$, $b_s = \sigma^s/V_\sigma$. If $\xi^{(s+1)}$ <0 then σ^* is sampled from a $N(\sigma^{(s)}, V_\sigma)I(-\xi^{(s+1)}(M-u^{(s)}, \infty)$ distribution. Therefore, $\sigma^{(s+1)} = \sigma^*$ with acceptance probability α_σ:

$$\alpha_\sigma = \min\left(1, \frac{p(\theta^*|x)g(\sigma^{(s)}|a_s, b_s)}{p(\tilde{\theta}|x)g(\sigma^*|a^*, b^*)}\right) \qquad \text{if } \xi^{(s+1)} \geq 0$$

$$\alpha_\sigma = \min\left(1, \frac{p(\theta^*|x)\Phi((\sigma^{(s)} + \xi^{(s+1)}(M-u^{(s)}))/\sqrt{V_\sigma})}{p(\tilde{\theta}|x)\Phi((\sigma^* + \xi^{(s+1)}/(M-u^{(s)}))/\sqrt{V_\sigma})}\right) \qquad \text{if } \xi^{(s+1)} < 0$$

1.3 Sampling u

u^* is sampled from a $N(u^{(s)}, V_u)I(a^{(s+1)}, M)$ distribution, where

$$a^{(s+1)} = \begin{cases} \min(x_1, ..., x_n), & \text{if } \xi^{(s+1)} \geq 0 \\ M + \sigma^{(s+1)}/\xi^{(s+1)}, & \text{if } \xi^{(s+1)} < 0 \end{cases} \tag{2}$$

Therefore $u^{(s+1)} = u^*$ with probability α_u where

$$\alpha_\xi = \min\left(1, \frac{p(\theta^*|x)\Phi((M-u^{(s)})/\sqrt{V_u}) - \Phi((a^{(s+1)} - u^{(s)})\sqrt{V_u})}{p(\tilde{\theta}|x)\Phi((M-u^*)/\sqrt{V_u}) - \Phi((a^{(s+1)} - u^*)\sqrt{V_u})}\right)$$

1.4 Sampling α and β

α^* and β^* are sampled, respectively, from $N(\alpha^{(s)}, V_\alpha)I(0.1, \infty)$, $N(\beta^{(s)}, V_\beta)I(0.1, \infty)$. Therefore, $\alpha^{(s+1)}$, $\beta^{(s+1)} = (\alpha^*$, β^* with probability

$$\min\left(1, \frac{p(\theta^*|x)h(\alpha^{(s)}|\alpha^*, V_\alpha)g(\beta^{(s+1)}|a_s, b_s)}{p(\tilde{\theta}|x)h(\alpha^*|\alpha^{(s)}, V_\alpha)g(\beta^*|a^*, b^*))}\right)$$

2 Model 2 & 3: Constant bulk regression with time-varying generalized pareto distribution

Below the threshold u, we place the following regression structure on Y, X the dependent and independent variables respectively, β the regression parameters of interest and white noise parameterized by ϵ. n denotes the number of observations below the threshold u and p the number of dependent variables in the regression equation:

$$\mathbf{Y}_{n\times 1} = \mathbf{X}_{n\times p}\beta_{p\times 1} + \epsilon_{n\times 1} \tag{3}$$
$$\epsilon \sim N(0, \sigma^2)$$

We estimate sequentially $\Theta_{-u} = \{\boldsymbol{\beta}, \sigma^2\}$ by placing the following priors on parameters:

$$\boldsymbol{\beta} \sim N\left(\boldsymbol{\beta_0}, \mathbf{P}_0\right)$$

with $\boldsymbol{\beta_0} = \mathbf{0}_{p\times 1}$ and $\mathbf{P}_0 = \text{diag}(100)_{p\times p}$ for our parameters to be centered around 0 and having a wide variance to impose noninformativeness with scalar $T_0 = \theta_0 = 1$ to yield a non-informative inverse-gamma prior distribution for σ.:

$$\sigma^2 \sim IG\left(\frac{T_0}{2}, \frac{\theta_0}{2}\right)$$

Above the threshold u, we place the generalized pareto distribution (GPD) with time varying parameters:

$$G(y_t; u, \Theta_u) = \begin{cases} 1 - \left(1 + \frac{\xi_t(y_t-u)}{\sigma_t}\right)^{-1/\xi_t} & \text{if } \xi_t \neq 0 \\ 1 - \exp(-(y_t-u)/\sigma_t) & \text{if } \xi_t = 0 \end{cases} \tag{4}$$

The GPD parameters ξ_t, σ_t follow random walk state equations with white noise as follows:

$$\xi_t = \xi_{t-1} + w_{\xi,t} \qquad w_{\xi,t} \sim N(0, 1/W_\xi)$$

$$\sigma_t = \sigma_{t-1} + w_{\sigma,t} \qquad w_{\sigma,t} \sim N(0, 1/W_\sigma)$$

We log-transform ξ_t, σ_t to allow parameters to be within the allowable bounds for the GPD ($\xi < -1$) with $l\xi_t = log(\xi_t + 1), l\sigma_t = log\sigma_t$:

$$l\xi_t = \theta_{\xi,t} + v_{\xi,t} \qquad v_{\xi,t} \sim N(0, 1/V_\xi)$$
$$l\sigma_t = \theta_{\sigma,t} + v_{\sigma,t} \qquad v_{\sigma,t} \sim N(0, 1/V_\sigma)$$
$$\theta_{\xi,t} = \theta_{\xi,t-1} + w_{\xi,t} \qquad w_{\xi,t} \sim N(0, 1/W_\xi)$$
$$\theta_{\sigma,t} = \theta_{\sigma,t-1} + w_{\sigma,t} \qquad w_{\sigma,t} \sim N(0, 1/W_\sigma)$$

We estimate sequentially $\{V_\xi, V_\sigma, W_\xi, W_\sigma\}$ by placing the following priors on parameters:

$$V_\xi \sim \text{Ga}(l_\xi, m_\xi)$$

$$V_\sigma \sim \text{Ga}(l_\sigma, m_\sigma)$$
$$W_\xi \sim \text{Ga}(f_\xi, o_\xi)$$
$$W_\sigma \sim \text{Ga}(f_\sigma, o_\sigma)$$

With the initial information for $\theta_{\xi,t}, \theta_{\sigma,t}$ given by:

$$\theta_{\sigma,0} \sim N(\mu_{\sigma,0}, C_{\sigma,0})$$

$$\theta_{\xi,0} \sim N(\mu_{\xi,0}, C_{\xi,0})$$

We apply the same threshold prior as Model 1.

2.1 Sampling $\boldsymbol{\beta}$ and σ^2

$\boldsymbol{\beta}$ is sampled from:

$$\boldsymbol{\beta}^* \sim (\Sigma_0^{-1} + \frac{1}{\sigma^2}X'X)^{-1}(\Sigma_0^{-1}\boldsymbol{\beta_0} + \frac{1}{\sigma^2}X'Y)$$

σ^2 is sampled from:

$$\sigma^2 \sim (\Sigma_0^{-1} + \frac{1}{\sigma^2}X'X)^{-1}$$

2.2 Sampling $\{l\xi_t\}_{t=1}^T$

If $x_t < u^{(s)}$, we sample parameters from $l\xi_t^{(s+1)} \sim N(\theta_{\xi,t}^{(s)}, 1/V_\xi^{(s)})$. If $x_t \geq u^{(s)}$, $l\xi_t^*$ is sampled from $N(l\xi_t^{(s)}, K_\xi)$. Therefore, $l\xi_t^{(s+1)} = l\xi_t^*$ with acceptance probability:

$$\min\left(1, \frac{p(\theta^*|y)}{p(\tilde{\theta}|y}\right)$$

2.3 Sampling $\{l\sigma_t\}_{t=1}^T$

If $x_t < u^{(s)}$, then the parameter can be sampled by: $l\sigma_t^{(s+1)} \sim N(\theta_{\sigma,t}^{(s)}, 1/V_\sigma^{(s)})$. If $x_t \geq u^{(s)}$, $l\sigma_t^*$ is sampled from $N(l\sigma_t^{(s)}, K_\sigma)$. Therefore, $l\sigma_t^{(s+1)} = l\sigma_t^*$ with probability

$$\min\left(1, \frac{p(\theta^*|y)}{p(\tilde{\theta}|y)}\right)$$

2.4 Sampling u

u^* is sampled from $N(u^{(s)}, V_u)I(u_L^{(s)}, \infty)$, where $u_L^{(s)} = \max\left(\min(x_{1:t}), \max_{\xi_t^{s+1}<0, x_t>u^s} \left(x_{1:t} + \sigma^{s+1}\right)/(\xi_t^{s+1}(1+\xi_t^{s+1})\right)$. Therefore, $u^{(s+1)}$ is accepted with probability:

$$\min\left(1, \frac{\pi(\Theta^*|x,y)\Phi((u^s - u_L^s)/\sqrt{V_u})}{\pi(\Theta|x,y)\Phi((u^s - u_L^s)/\sqrt{V_u})}\right)$$

$\{V_\xi,\, W_\xi,\, \theta_{\xi,t},\, V_\sigma,\, W_\sigma,\, \theta_{\sigma,t}\}$ are updated via the following Gibbs steps:

$$V_\xi^{(s+1)} \sim G\left(f_\xi + \frac{T}{2}, o_\xi + \frac{1}{2}\sum_{t=1}^{T}(l\xi_t^{(s+1)} - \theta_{\xi,t}^{(s)})^2\right)$$

$$W_\xi^{(s+1)} \sim G\left(l_\xi + \frac{T}{2}, m_\xi + \frac{1}{2}\sum_{t=1}^{T}(l\theta_{\xi,t}^(s) - \theta_{\xi,t-1}^{(s)})^2\right)$$

$$\theta_{\xi,0}^{(s+1)} \sim N\left(\frac{W_\xi^{(s+1)}\theta_{\xi,1}^{(s)} + m_{\xi,0}/C_{\xi,0}}{W_\xi^{(s+1)} + 1/C_{\xi,0}}, \frac{1}{W_\xi^{(s+1)} + 1/C_{\xi,0}}\right)$$

$$\theta_{\xi,t}^{(s+1)} \sim N\left(\frac{V_\xi^{(s+1)}l\xi_t^{(s+1)} + W_\xi^{(s+1)}(\theta_{\xi,t+1}^{(s)} + \theta_{\xi,t-1}^{(s+1)})}{V_\xi^{(s+1)} + 2W_\xi^{(s+1)}}, \frac{1}{V_\xi^{(s+1)} + 2W_\xi^{(s+1)}}\right)$$

$$\theta_{\xi,T}^{(s+1)} \sim N\left(\frac{V_\xi^{(s+1)}l\xi_T^{(s+1)} + W_\xi^{(s+1)}\theta_{\xi,T-1}^{(s+1)}}{V_\xi^{(s+1)} + W_\xi^{(s+1)}}, \frac{1}{V_\xi^{(s+1)} + W_\xi^{(s+1)}}\right)$$

Posterior distributions for V_σ, W_σ, $\theta_{\sigma,t}$ follow the same functional form.

3 Model 4: Constant bulk regression and generalized pareto distribution regression

We apply the same bulk distribution and extreme value distribution as Model 2 and 3, but additionally impose regression structure for the extreme parameters for Model 4. We set the following priors for $\boldsymbol{\beta_\xi}$ and $\boldsymbol{\beta_\sigma}$:

$$\boldsymbol{\beta} \sim N(\boldsymbol{\beta_0}, 1/\Sigma)$$

where $\boldsymbol{\beta_0} = \mathbf{0}_{p\times 1}, \Sigma = \text{diag}(\frac{1}{100})_{p\times p}$

$$l\xi_t = \boldsymbol{\beta}_{\xi,t} + v_{\xi,t} \qquad v_{\xi,t} \sim N(0, 1/V_\xi)$$

$$l\sigma_t = \boldsymbol{\beta}_{\sigma,t} + v_{\sigma,t} \qquad v_{\sigma,t} \sim N(0, 1/V_\sigma)$$

where $\boldsymbol{\beta}_{\xi,t} = \sum_{k=0}^{p}\beta_{\xi,k}X_{t,k}$, $\boldsymbol{\beta}_{\sigma,t} = \sum_{k=0}^{p}\beta_{\sigma,k}X_{t,k}$

3.1 Sampling β and σ^2

$\boldsymbol{\beta}$ is sampled from

$$\boldsymbol{\beta}^* \sim (\Sigma_0^{-1} + \frac{1}{\sigma^2}X'X)^{-1}(\Sigma_0^{-1}\boldsymbol{\beta_0} + \frac{1}{\sigma^2}X'Y)$$

σ^2 is sampled from

$$\sigma^2 \sim (\Sigma_0^{-1} + \frac{1}{\sigma^2}X'X)^{-1}$$

3.2 Sampling $\{l\xi_t\}_{t=1}^T$

If $x_t < u^{(s)}$, then the parameter can be sampled by: $l\xi_t^{(s+1)} \sim N(\boldsymbol{\beta}_{\xi,t}^{(s)}, 1/V_\xi^{(s)})$. If $x_t \geq u^{(s)}$, $l\xi_t^*$ is sampled from $N(l\xi_t^{(s)}, K_\xi)$. Therefore, $l\xi_t^{(s+1)} = l\xi_t^*$ with probability

$$\min\left(1, \frac{p(\theta^*|y)}{p(\tilde{\theta}|y}\right)$$

where $\boldsymbol{\beta}_{\xi,t} = \sum_{k=0}^{p}\beta_{\xi,t,k}X_{t,k}$.

3.3 Sampling $\{l\sigma_t\}_{t=1}^T$

If $x_t < u^{(s)}$, then the parameter can be sampled by: $l\sigma_t^{(s+1)} \sim N(\boldsymbol{\beta}_{\sigma,t}^{(s)}, 1/V_\sigma^{(s)})$. If $x_t \geq u^{(s)}$, $l\sigma_t^*$ is sampled from $N(l\sigma_t^{(s)}, K_\sigma)$. Therefore, $l\sigma_t^{(s+1)} = l\sigma_t^*$ with probability

$$\min\left(1, \frac{p(\theta^*|y)}{p(\tilde{\theta}|y)}\right)$$

where $\boldsymbol{\beta}_{\sigma,t} = \sum_{k=0}^p \beta_{\xi,t,k} X_{t,k}$.

3.4 Sampling u

u^* is sampled from a $N(u^{(s)}, V_u)I(u_L^{(s)}, \infty)$, where $u_L^{(s)} = \max\left(\min(x_{1:t}), \max_{\xi_t^{s+1}<0, x_t>u^s} \left(x_{1:t} + \sigma^{s+1}\right)/(\xi_t^{s+1}(1+\xi_t^{s+1})\right)$. Therefore, $u^{(s+1)}$ is accepted with probability:

$$min\left(1, \frac{p(\theta^*|x,y)\Phi((u^s - u_L^s)/\sqrt{V_u})}{p(\theta|x,y)\Phi((u^s - u_L^s)/\sqrt{V_u})}\right)$$

$\{V_\xi, \boldsymbol{\beta}_\xi, V_\sigma, \boldsymbol{\beta}_\sigma\}$ can be updated via Gibbs steps.

$$V_\xi^{(s+1)} \sim G\left(f_\xi + \frac{T}{2}, o_\xi + \frac{1}{2}\sum_{t=1}^T (l\xi_t^{(s+1)} - \boldsymbol{\beta}_{\xi,t}^{(s)})^2\right)$$

$$\boldsymbol{\beta}_\xi \sim N\left(\frac{X'V_\xi \boldsymbol{l\xi} + \Sigma\boldsymbol{\beta_0}}{X'V_\xi X + \Sigma}, \frac{1}{X'V_\xi X + \Sigma}\right)$$

where $\boldsymbol{l\xi} = \{l\xi_t\}_{t=1}^T$. Posterior distributions for V_σ,$\boldsymbol{\beta}_\sigma$ also follow the same functional form.

4 Model 5: Constant bulk regression and time-varying generalized pareto regression

In Model 4, the coefficients are constant in the regression structure. We use linear dynamic model which allows time-varying coefficients in Model 5.

$$l\xi_t = \boldsymbol{\beta}_{\xi,t} + v_{\xi,t} \qquad v_{\xi,t} \sim N(0, 1/V_\xi)$$

$$\boldsymbol{\beta}_{\xi,t,k} = \beta_{\xi,t-1,k} + \omega_{\xi,t} \qquad \omega_{\xi,t} \sim N(0, 1/W_{\xi,k})$$

$$l\sigma_t = \boldsymbol{\beta}_{\sigma,t} + v_{\sigma,t} \qquad v_{\sigma,t} \sim N(0, 1/V_\sigma)$$

$$\boldsymbol{\beta}_{\sigma,t,k} = \beta_{\sigma,t-1,k} + \omega_{\sigma,t} \qquad \omega_{\sigma,t} \sim N(0, 1/W_{\sigma,k})$$

where $\boldsymbol{\beta}_{\xi,t} = \sum_{k=0}^p \beta_{\xi,t,k} X_{t,k}$, $\boldsymbol{\beta}_{\sigma,t} = \sum_{k=0}^p \beta_{\sigma,t,k} X_{t,k}$

4.1 Sampling β and σ^2

$\boldsymbol{\beta}$ is sampled from

$$\boldsymbol{\beta}^* \sim (\Sigma_0^{-1} + \frac{1}{\sigma^2}X'X)^{-1}(\Sigma_0^{-1}\boldsymbol{\beta_0} + \frac{1}{\sigma^2}X'Y)$$

σ^2 is sampled from

$$\sigma^2 \sim (\Sigma_0^{-1} + \frac{1}{\sigma^2}X'X)^{-1}$$

4.2 Sampling $\{l\xi_t\}_{t=1}^T$

If $x_t < u^{(s)}$, then the parameter can be sampled by: $l\xi_t^{(s+1)} \sim N(\boldsymbol{\beta}_{\xi,t}^{(s)}, 1/V_\xi^{(s)})$. If $x_t \geq u^{(s)}$, $l\xi_t^*$ is sampled from $N(l\xi_t^{(s)}, K_\xi)$. Therefore, $l\xi_t^{(s+1)} = l\xi_t^*$ with probability

$$\min\left(1, \frac{p(\theta^*|y)}{p(\tilde{\theta}|y}\right)$$

where $\boldsymbol{\beta}_{\xi,t} = \sum_{k=0}^p \beta_{\xi,t,k} X_{t,k}$.

4.3 Sampling $\{l\sigma_t\}_{t=1}^T$

If $x_t < u^{(s)}$, then the parameter can be sampled by: $l\sigma_t^{(s+1)} \sim N(\boldsymbol{\beta}_{\sigma,t}^{(s)}, 1/V_\sigma^{(s)})$. If $x_t \geq u^{(s)}$, $l\sigma_t^*$ is sampled from $N(l\sigma_t^{(s)}, K_\sigma)$. Therefore, $l\sigma_t^{(s+1)} = l\sigma_t^*$ with probability

$$\min\left(1, \frac{p(\theta^*|y)}{p(\tilde{\theta}|y)}\right)$$

where $\boldsymbol{\beta}_{\sigma,t} = \sum_{k=0}^p \beta_{\xi,t,k} X_{t,k}$.

4.4 Sampling u

u^* is sampled from a $N(u^{(s)}, V_u)I(u_L^{(s)}, \infty)$, where $u_L^{(s)} = \max\left(\min(x_{1:t}), \max_{\xi_t^{s+1}<0, x_t>u^s} \left(x_{1:t} + \sigma^{s+1}\right)/(\xi_t^{s+1}(1+\xi_t^{s+1})\right)$. Therefore, $u^{(s+1)}$ is accepted with probability:

$$\min\left(1, \frac{p(\theta^*|x,y)\Phi((u^s - u_L^s)/\sqrt{V_u})}{p(\theta|x,y)\Phi((u^s - u_L^s)/\sqrt{V_u})}\right)$$

$\{V_\xi,\ W_{\xi,k},\ \beta_{\xi,t,k},\ V_\sigma,\ W_{\sigma,k},\ \beta_{\sigma,t,k}\}$ can be updated via Gibbs steps.

$$V_\xi^{(s+1)} \sim G\left(f_\xi + \frac{T}{2}, o_\xi + \frac{1}{2}\sum_{t=1}^T (l\xi_t^{(s+1)} - \boldsymbol{\beta}_{\xi,t}^{(s)})^2\right)$$

$$W_{\xi,k}^{(s+1)} \sim G\left(l_\xi + \frac{T}{2}, m_\xi + \frac{1}{2}\sum_{t=1}^T (\beta_{\xi,t,k}^{(s)} - \beta_{\xi,t-1,k}^{(s)})^2\right)$$

$$\beta_{\xi,0,k}^{(s+1)} \sim N\left(\frac{W_{\xi,k}^{(s+1)}\beta_{\xi,1,k}^{(s)} + m_{\xi,0}/C_{\xi,0}}{W_{\xi,k}^{(s+1)} + 1/C_{\xi,0}}, \frac{1}{W_{\xi,k}^{(s+1)} + 1/C_{\xi,0}}\right)$$

$$\beta_{\xi,t,k}^{(s+1)} \sim N\left(\frac{V_\xi^{(s+1)}S_{t,k}X_{t,k} + W_{\xi,k}^{(s+1)}(\beta_{\xi,t+1,k}^{(s)} + \beta_{\xi,t-1,k}^{(s+1)})}{V_\xi^{(s+1)}X_{t,k}^2 + 2W_{\xi,k}^{(s+1)}}, \frac{1}{V_\xi^{(s+1)}X_{t,k}^2 + 2W_{\xi,k}^{(s+1)}}\right)$$

$$\beta_{\xi,T,k}^{(s+1)} \sim N\left(\frac{V_\xi^{(s+1)}S_{T,k}X_{T,k} + W_{\xi,k}^{(s+1)}\beta_{\xi,T-1,k}^{(s+1)}}{V_\xi^{(s+1)}X_{T,k}^2 + W_{\xi,k}^{(s+1)}}, \frac{1}{V_\xi^{(s+1)}X_{T,k}^2 + W_{\xi,k}^{(s+1)}}\right)$$

where $S_{t,k} = l\xi_t - \sum_{i=0,i\neq k}^p \beta_{\xi,t,i}X_{t,i}$. Posterior distributions for V_σ, $W_{\sigma,k}$, $\beta_{\sigma,t,k}$ also follow the same functional form.

5 Model Assessment

We use the deviation information criterion (DIC) and log Bayes factor (logBF) to assess the appriopriateness of each model for the data generating process.

$$DIC = 2 \times \bar{D(\theta)} - D(\bar{\theta}) \tag{5}$$

where $\bar{D(\theta)}$ is the average of D(θ) over all samples of θ, D($\bar{\theta}$) is the value of D evaluated at the average of the samples of θ.

$$D(\theta) = -2 \times log(p(y|\theta)) + C \tag{6}$$

C is a constant that can be ignored during the calculation.

$$BF = \frac{p(D|M_1)}{p(D|M_2)}$$

$$logBF = log(p(D|M_1)) - log(p(D|M_2)) \tag{7}$$

where $p(D|M)$ denotes the likelihood that some data is produced under the assumption of model M.
