# Supplementary material for: Time varying methods to infer extremes in dengue transmission dynamics
Source: PLoS Comput Biol. 2020 Oct 12;16(10):e1008279. doi: 10.1371/journal.pcbi.1008279 (PMC7595636; doi:10.1371/journal.pcbi.1008279)
Supplement: S1 Fig — (PDF) [file pcbi.1008279.s002.pdf]

Appendix 2

September 24, 2020

Contents

1 Convergence plots for all models **2**

2 Quantile-Quantile plots for all models **7**

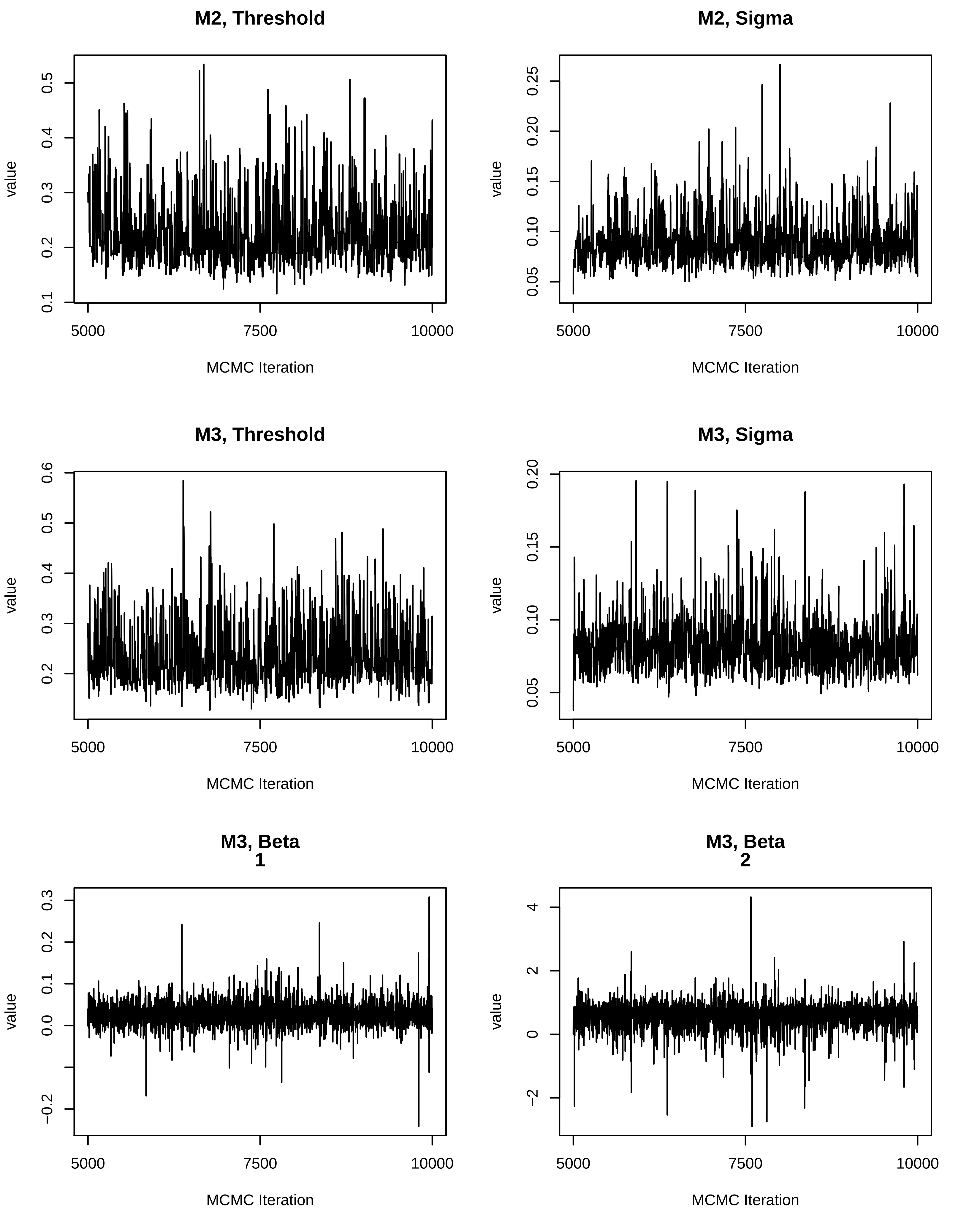
M2, Threshold
value
0.1
0.2
0.3
0.4
0.5
5000
7500
10000
MCMC Iteration
M2, Sigma
value
0.05
0.10
0.15
0.20
0.25
5000
7500
10000
MCMC Iteration
M3, Threshold
value
0.2
0.3
0.4
0.5
0.6
5000
7500
10000
MCMC Iteration
M3, Sigma
value
0.05
0.10
0.15
0.20
5000
7500
10000
MCMC Iteration
M3, Beta
1
value
−0.2
0.0
0.1
0.2
0.3
5000
7500
10000
MCMC Iteration
M3, Beta
2
value
−2
0
2
4
5000
7500
10000
MCMC Iteration

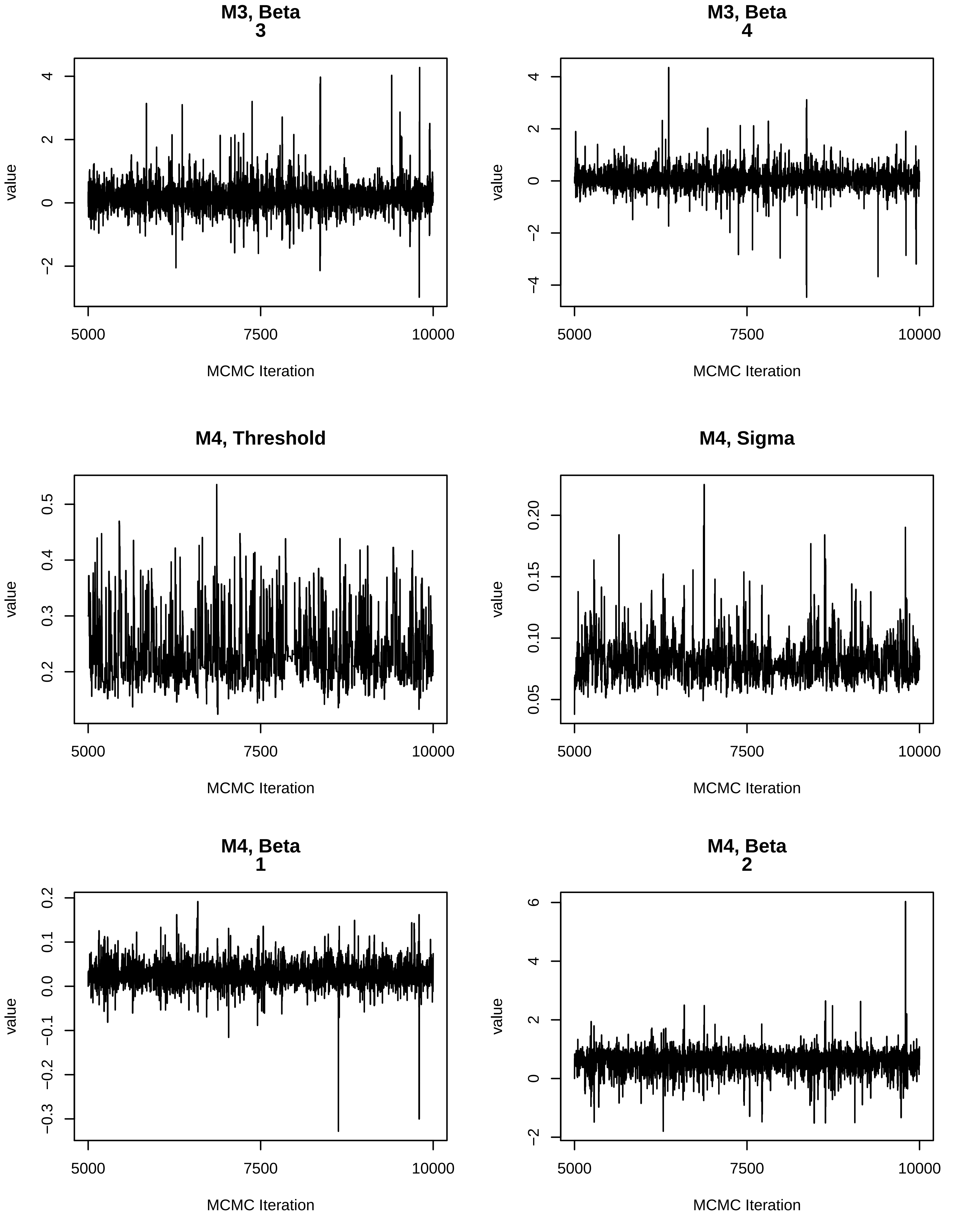
M3, Beta
3
value
MCMC Iteration
5000
7500
10000
−2
0
2
4
M3, Beta
4
value
MCMC Iteration
5000
7500
10000
−4
−2
0
2
4
M4, Threshold
value
MCMC Iteration
5000
7500
10000
0.2
0.3
0.4
0.5
M4, Sigma
value
MCMC Iteration
5000
7500
10000
0.05
0.10
0.15
0.20
M4, Beta
1
value
MCMC Iteration
5000
7500
10000
−0.3
−0.2
−0.1
0.0
0.1
0.2
M4, Beta
2
value
MCMC Iteration
5000
7500
10000
−2
0
2
4
6

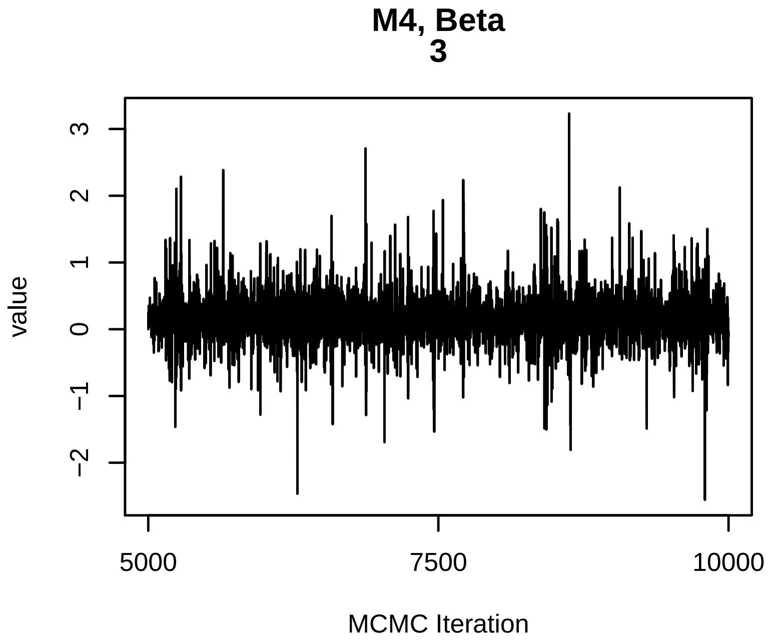
M4, Beta
3
value
MCMC Iteration
5000
7500
10000
−2
−1
0
1
2
3

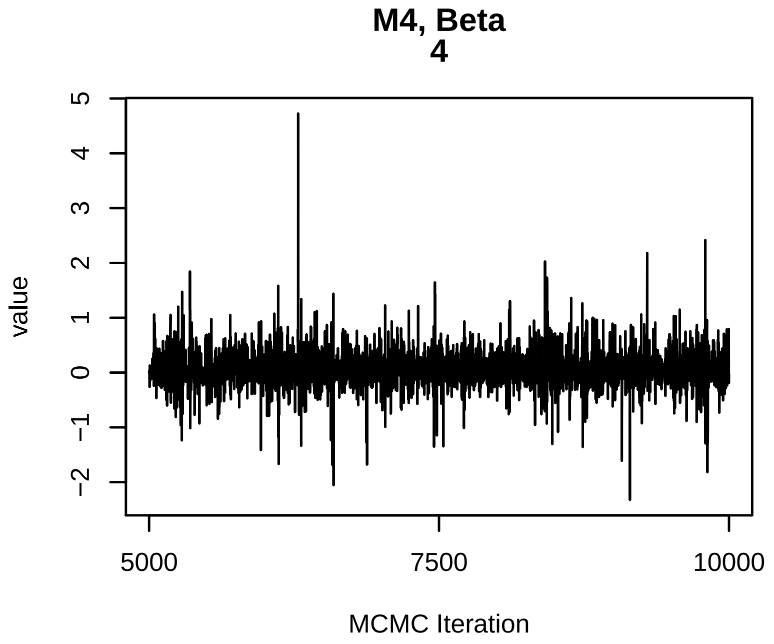
M4, Beta
4
value
MCMC Iteration
5000
7500
10000
−2
−1
0
1
2
3
4
5

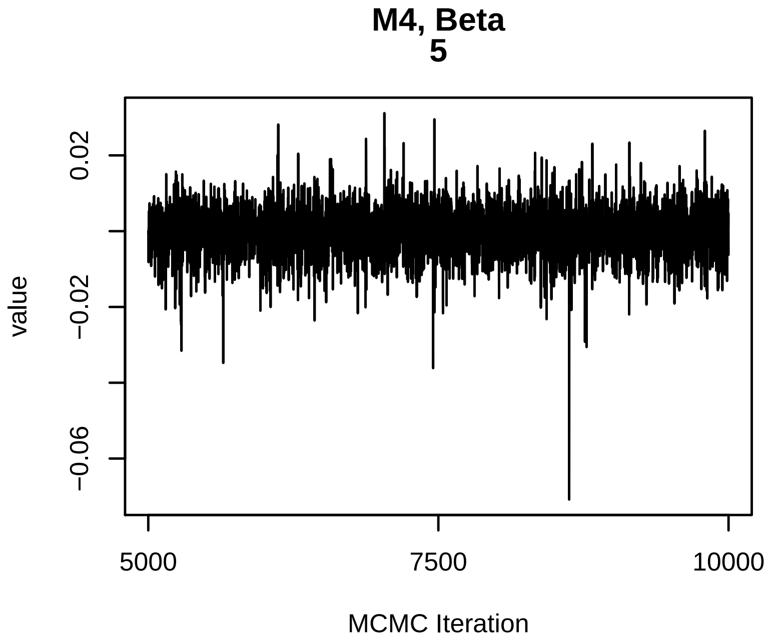
M4, Beta
5
value
MCMC Iteration
5000
7500
10000
−0.06
−0.02
0.02

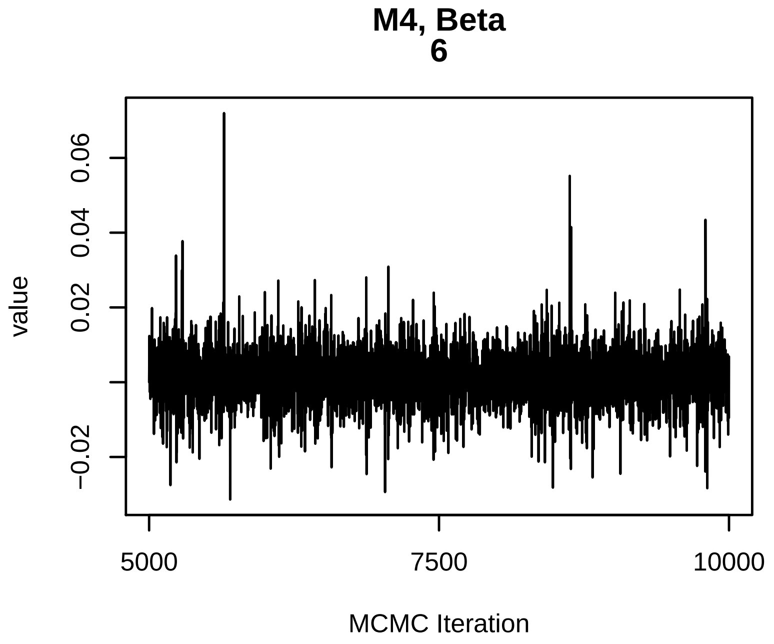
M4, Beta
6
value
MCMC Iteration
5000
7500
10000
−0.02
0.02
0.04
0.06

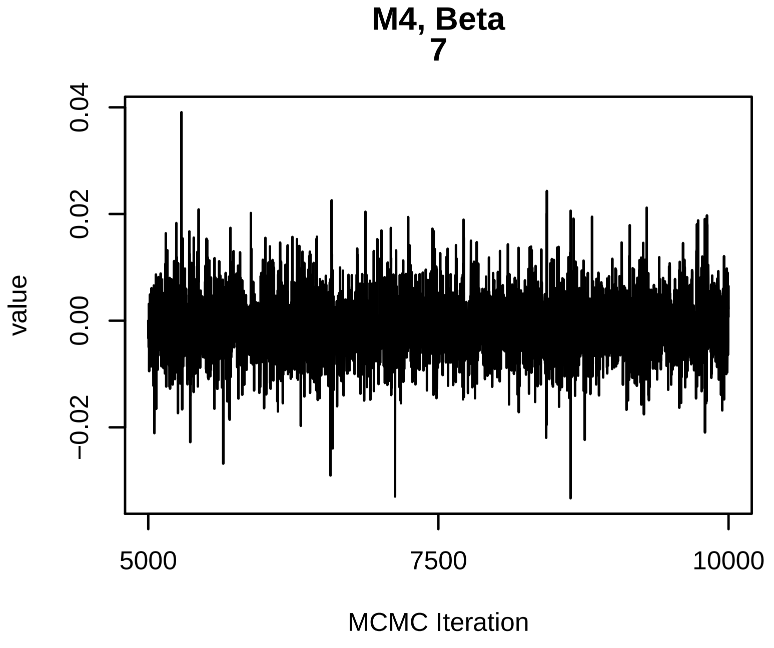
M4, Beta
7
value
MCMC Iteration
5000
7500
10000
−0.02
0.00
0.02
0.04

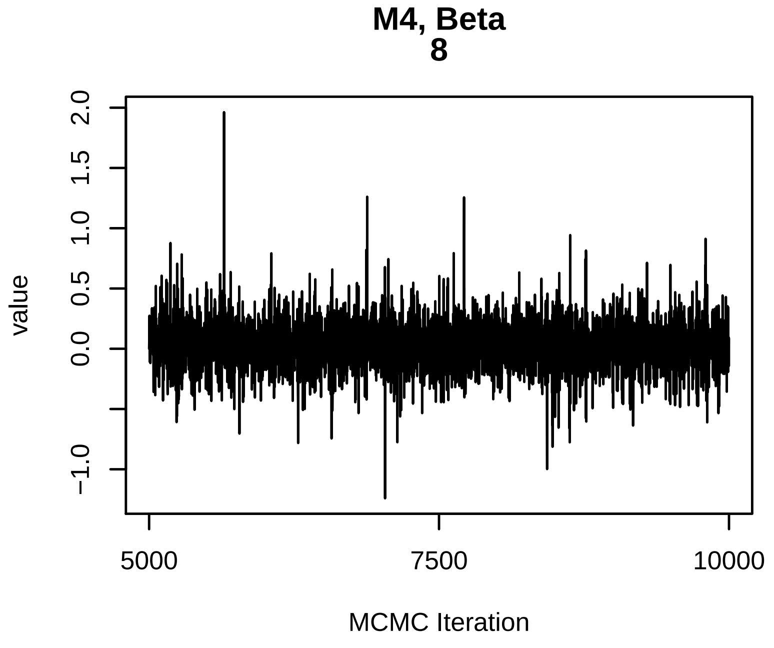
M4, Beta
8
value
MCMC Iteration
5000
7500
10000
−1.0
0.0
0.5
1.0
1.5
2.0

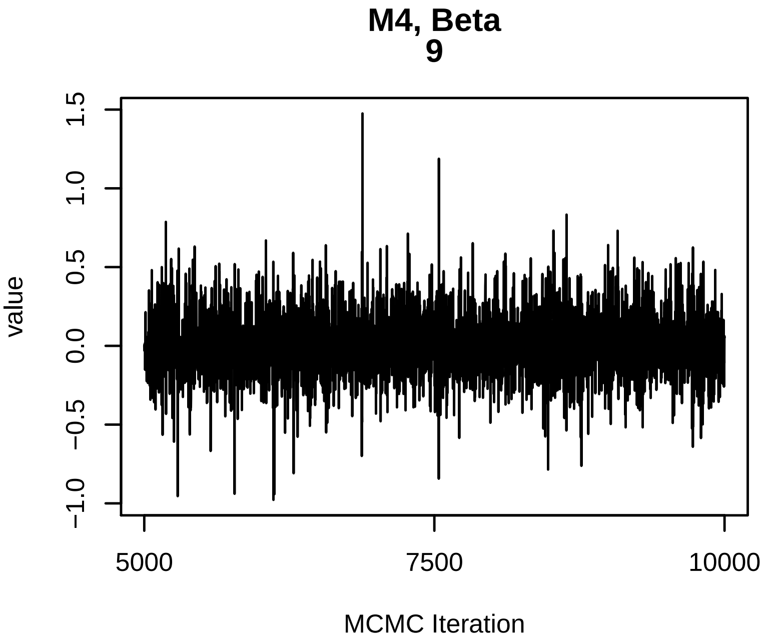
M4, Beta
9
value
1.5
1.0
0.5
0.0
−0.5
−1.0
5000
7500
10000
MCMC Iteration

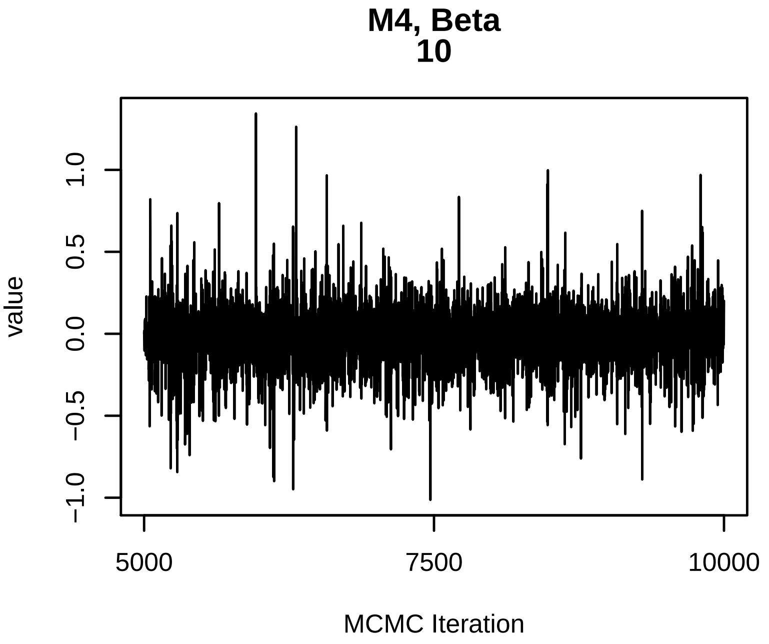
M4, Beta
10
value
1.0
0.5
0.0
−0.5
−1.0
5000
7500
10000
MCMC Iteration

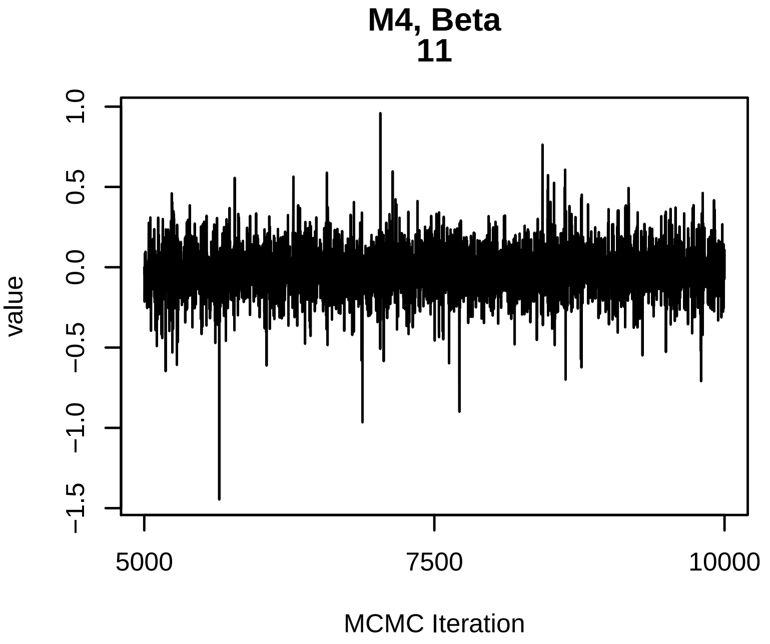
M4, Beta
11
value
1.0
0.5
0.0
−0.5
−1.0
−1.5
5000
7500
10000
MCMC Iteration

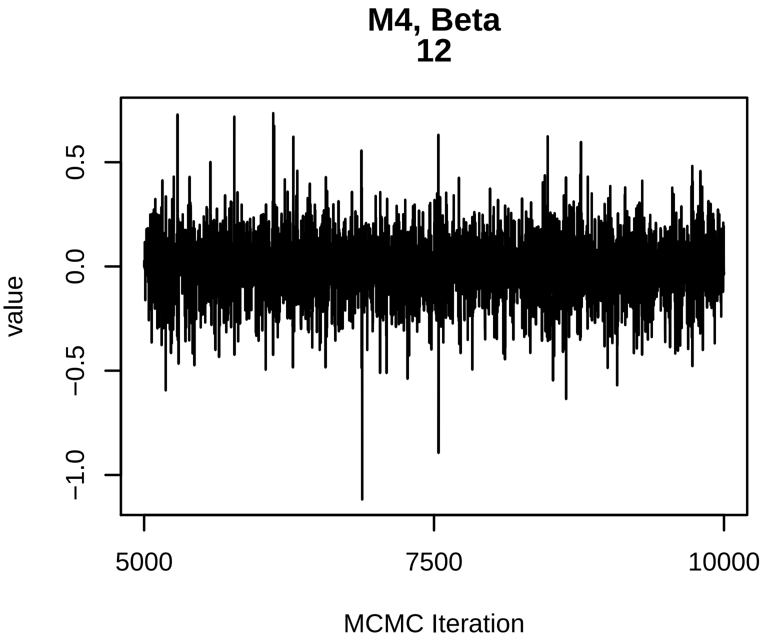
M4, Beta
12
value
0.5
0.0
−0.5
−1.0
5000
7500
10000
MCMC Iteration

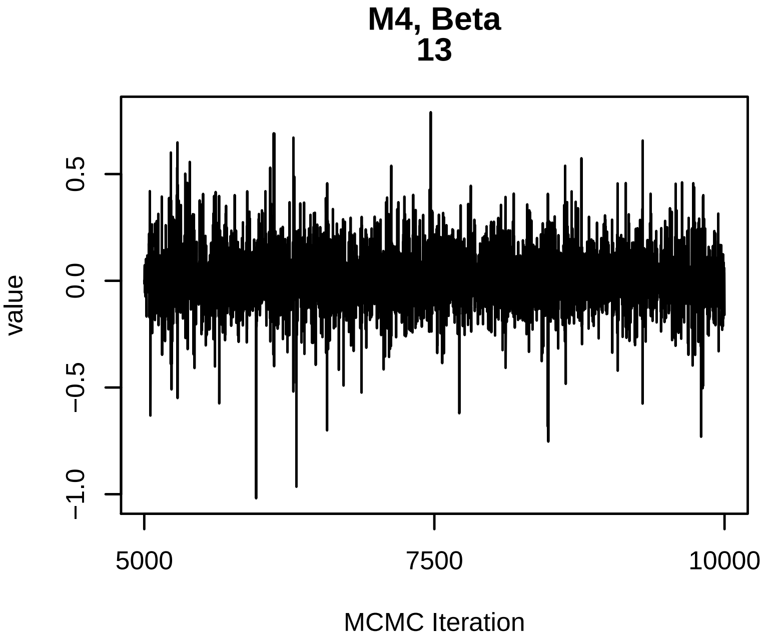
M4, Beta
13
value
0.5
0.0
−0.5
−1.0
5000
7500
10000
MCMC Iteration

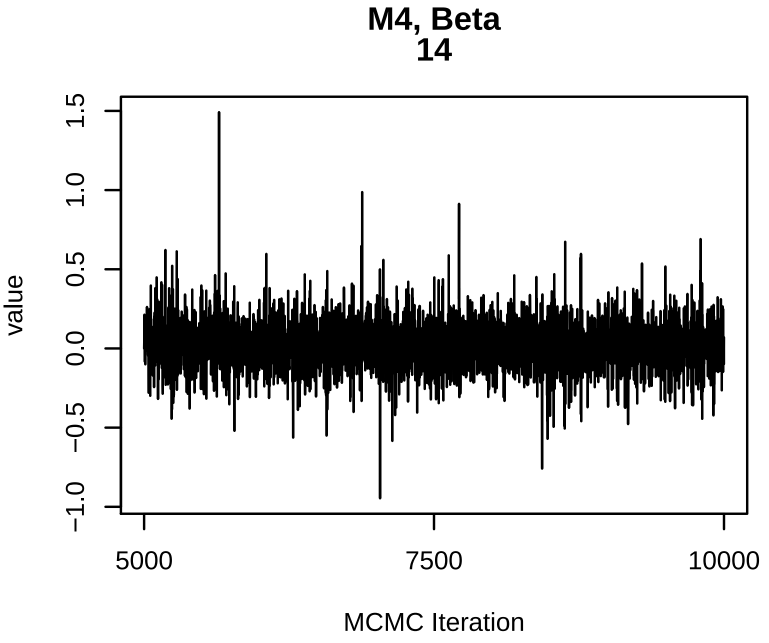
M4, Beta
14
value
1.5
1.0
0.5
0.0
−0.5
−1.0
5000
7500
10000
MCMC Iteration

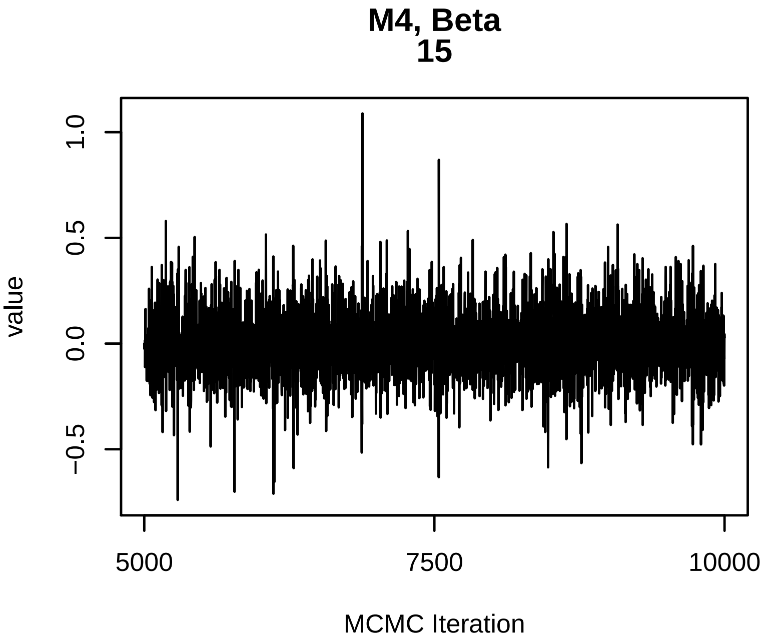
M4, Beta
15
value
1.0
0.5
0.0
−0.5
5000
7500
10000
MCMC Iteration

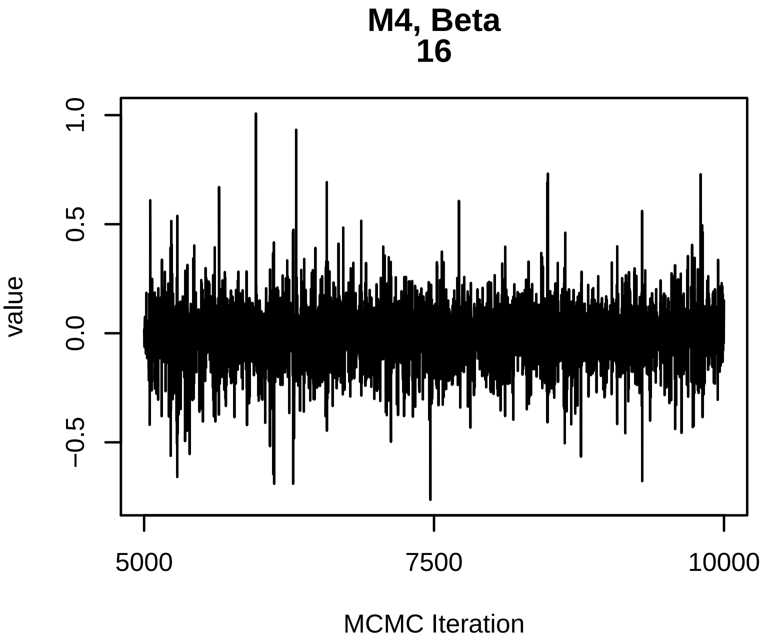
M4, Beta
16
value
1.0
0.5
0.0
−0.5
5000
7500
10000
MCMC Iteration

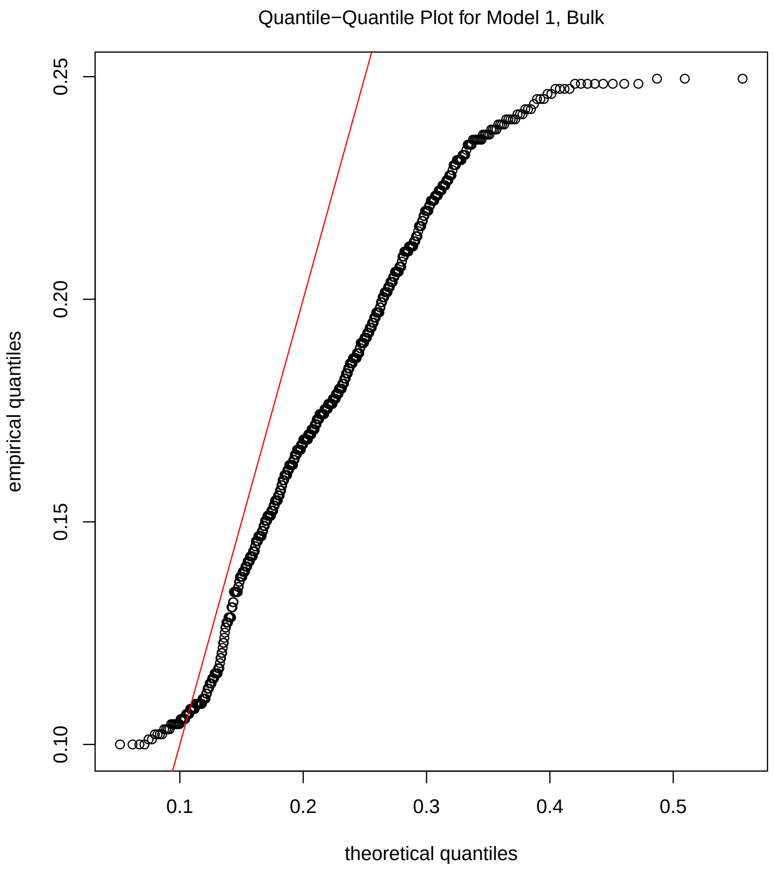
Quantile−Quantile Plot for Model 1, Bulk
empirical quantiles
theoretical quantiles
0.10
0.15
0.20
0.25
0.1
0.2
0.3
0.4
0.5

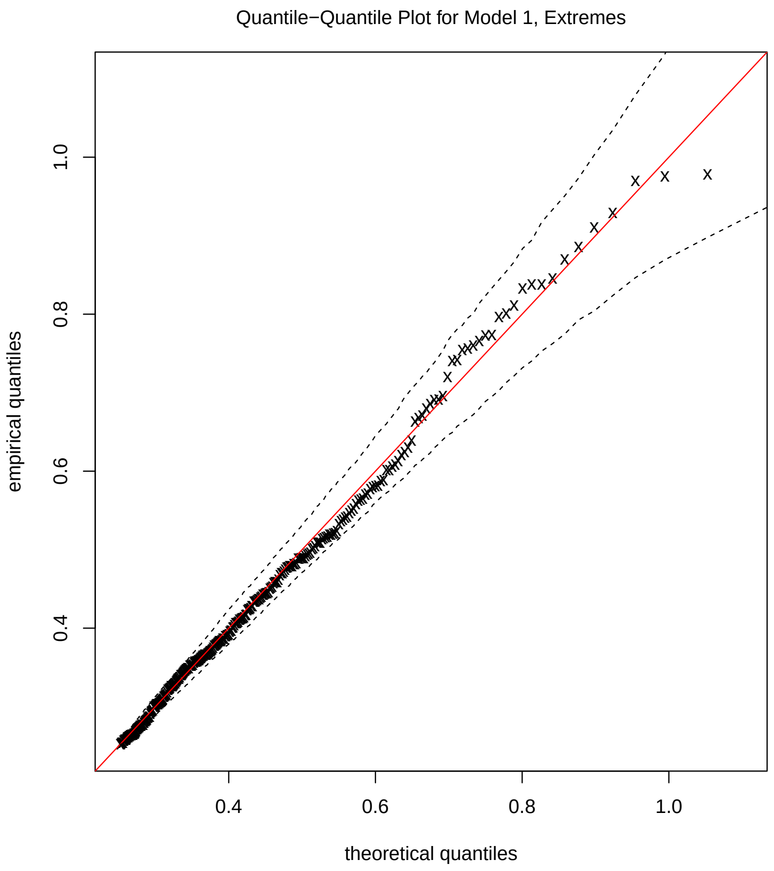
Quantile−Quantile Plot for Model 1, Extremes
empirical quantiles
theoretical quantiles
0.4
0.6
0.8
1.0
0.4
0.6
0.8
1.0

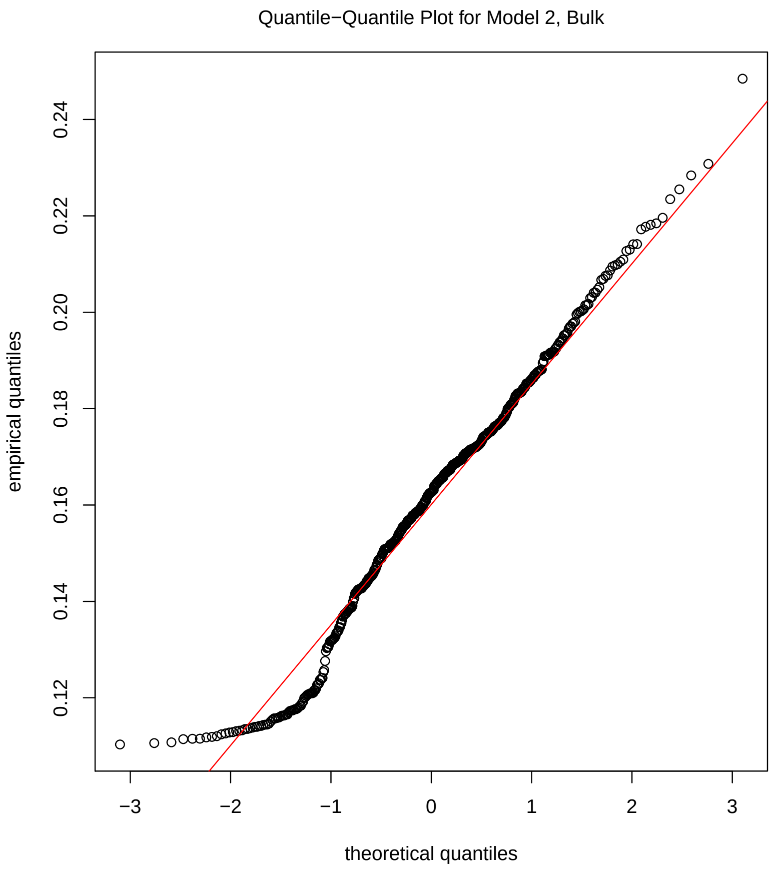

Quantile−Quantile Plot for Model 2, Bulk
empirical quantiles
theoretical quantiles
0.12
0.14
0.16
0.18
0.20
0.22
0.24
−3
−2
−1
0
1
2
3

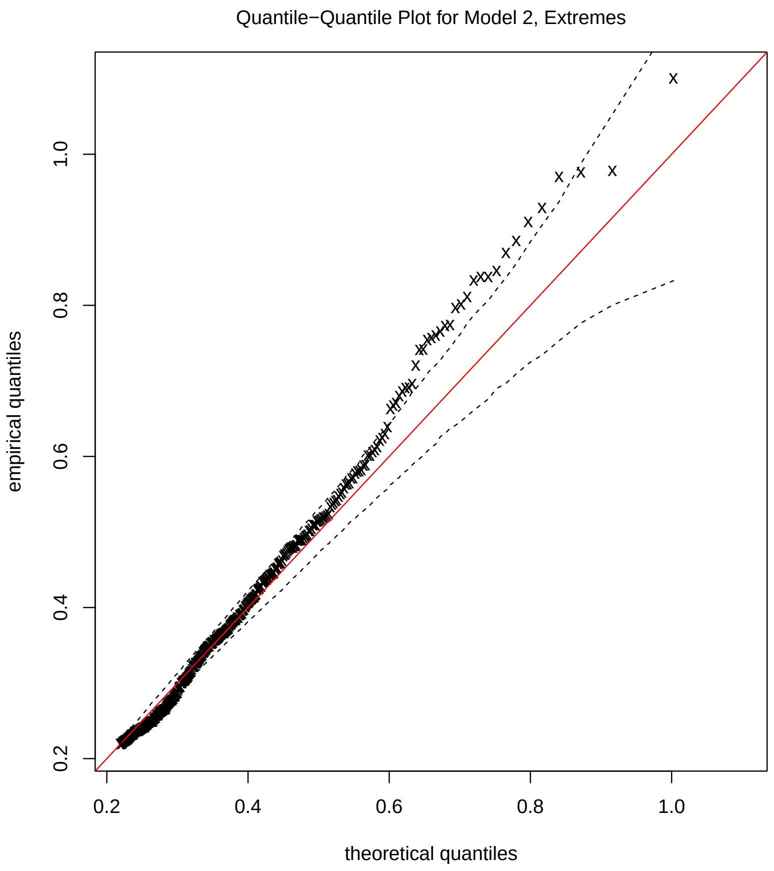

Quantile−Quantile Plot for Model 2, Extremes
empirical quantiles
theoretical quantiles
0.2
0.4
0.6
0.8
1.0
0.2
0.4
0.6
0.8
1.0

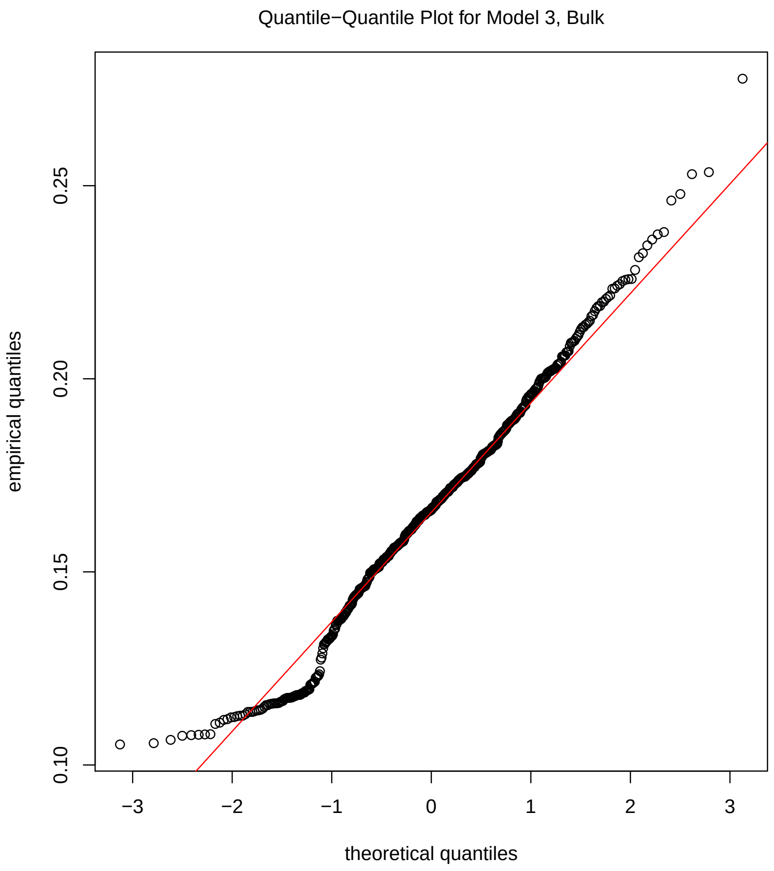

Quantile−Quantile Plot for Model 3, Bulk
empirical quantiles
theoretical quantiles
0.10
0.15
0.20
0.25
−3
−2
−1
0
1
2
3

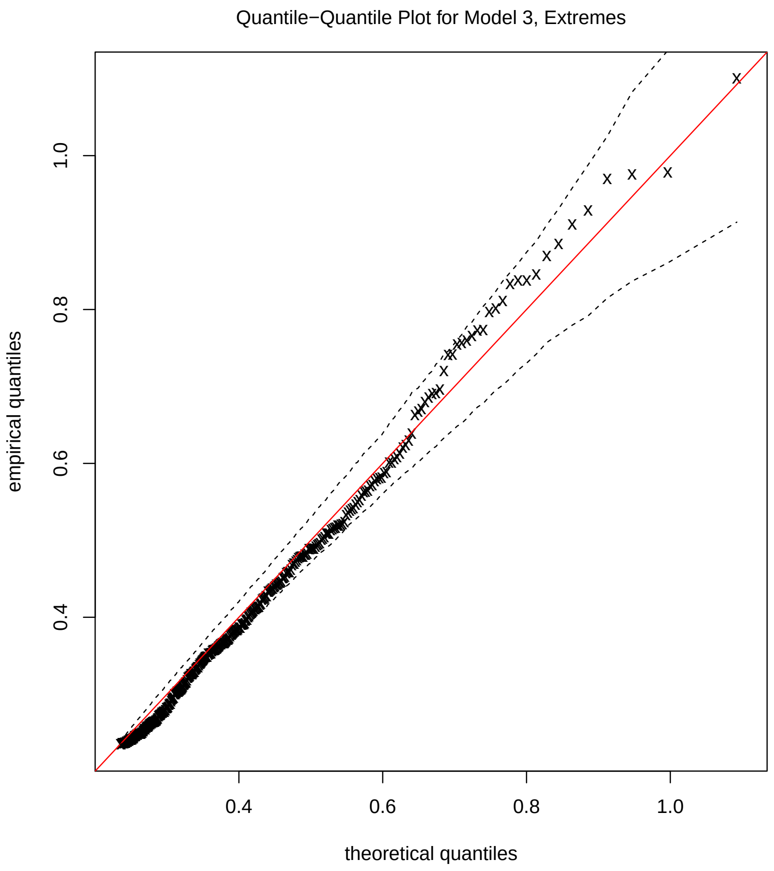

Quantile−Quantile Plot for Model 3, Extremes
empirical quantiles
theoretical quantiles
0.4
0.6
0.8
1.0
0.4
0.6
0.8
1.0

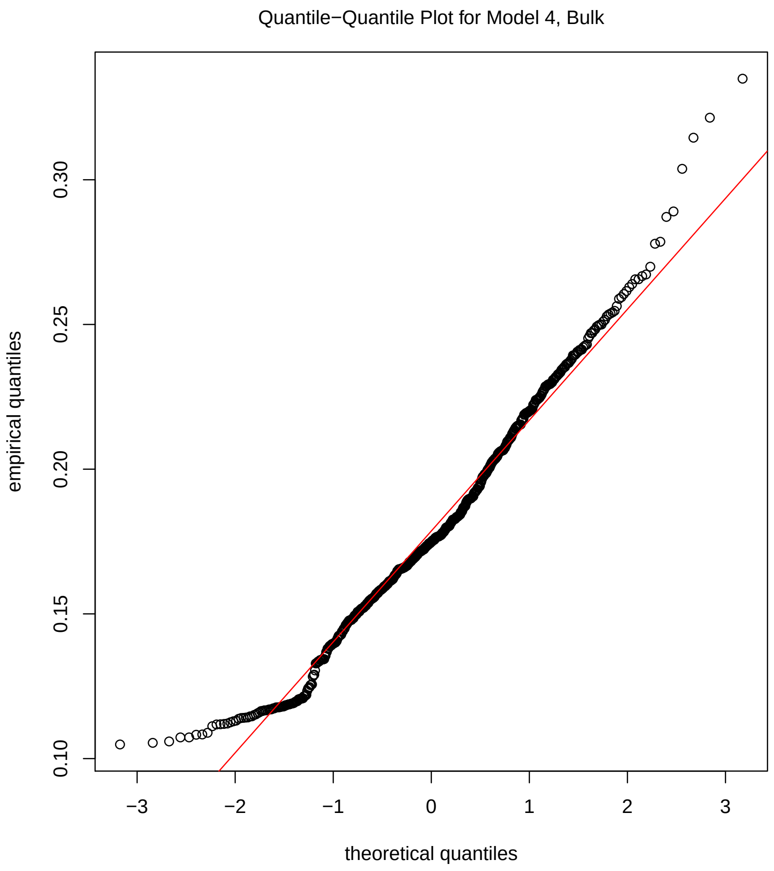
Quantile−Quantile Plot for Model 4, Bulk
empirical quantiles
theoretical quantiles
0.10
0.15
0.20
0.25
0.30
−3
−2
−1
0
1
2
3

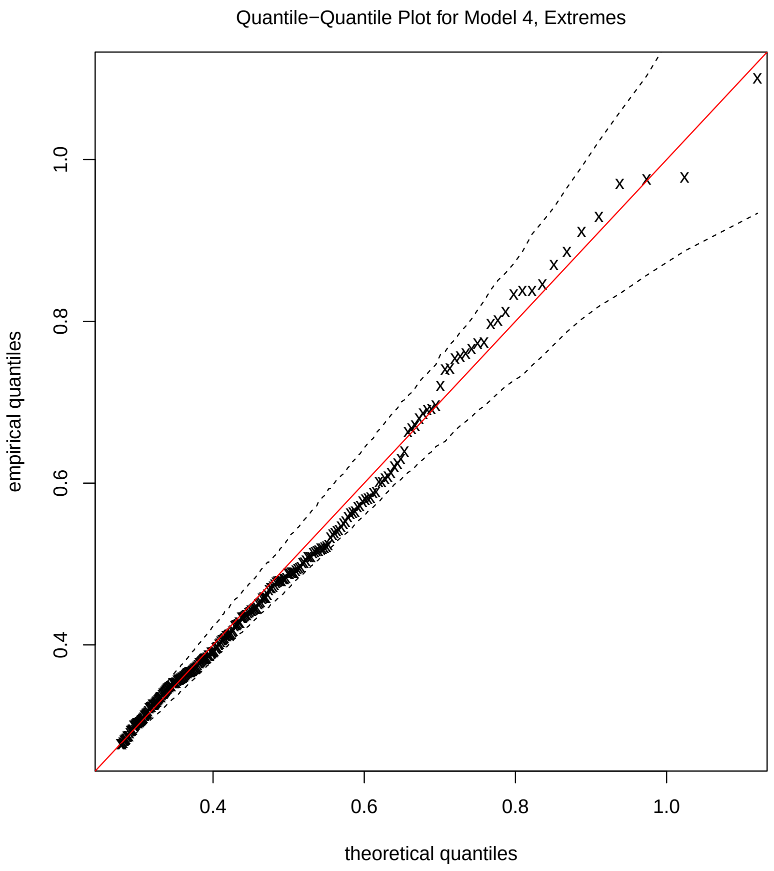
Quantile−Quantile Plot for Model 4, Extremes
empirical quantiles
theoretical quantiles
0.4
0.6
0.8
1.0
0.4
0.6
0.8
1.0
